# Unlocking the genetic diversity of Indian turmeric (*Curcuma longa* L.) germplasm based on rhizome yield traits and curcuminoids

**DOI:** 10.3389/fpls.2022.1036592

**Published:** 2022-12-15

**Authors:** Mastan Vali Dudekula, Venkatesan Kandasamy, Senthamizh Selvi Balaraman, Selva Babu Selvamani, Raveendran Muthurajan, Karthikeyan Adhimoolam, Bharani Manoharan, Senthil Natesan

**Affiliations:** ^1^ Department of Spices and Plantation Crops, Horticultural College and Research Institute, Tamil Nadu Agricultural University, Coimbatore, India; ^2^ Department of Plant Molecular Biology and Bioinformatics, Centre for Plant Molecular Biology and Biotechnology, Tamil Nadu Agricultural University, Coimbatore, Tamil Nadu, India; ^3^ Department of Plant Biotechnology, Centre for Plant Molecular Biology and Biotechnology, Tamil Nadu Agricultural University, Coimbatore, Tamil Nadu, India; ^4^ Department of Biotechnology, Centre of Innovation, Agricultural College and Research Institute, Tamil Nadu Agricultural University, Madurai, India; ^5^ Subtropical Horticulture Research Institute, Jeju National University, Jeju, South Korea

**Keywords:** Turmeric, curcuminoids, genetic diversity, germplasm, rhizome yield traits

## Abstract

Turmeric is an important commercial crop widely grown in Asia due to its pharmacological and nutritional value. India is the centre of turmeric diversity and many turmeric accessions have good rhizome yield, varying curcuminoids content and are well-adapted to various agro-climatic zones. In the present study, we unravel the diversity among 200 Indian turmeric accessions based on rhizome yield traits and curcuminoids content. Clustering and correlation studies were also performed to group the turmeric accessions and to observe the relationship between the traits. Results revealed the presence of large variability among turmeric accessions including the major traits such as yield (24.77 g p^-1^ to 667.63 g p^-1^), dry recovery percentage (13.42% to 29.18%), curcumin (0.41% to 2.17%), demethoxycurcumin (0.38% to 1.45%), bisdemethoxycurcumin (0.37% to 1.24%) and total curcuminoid content (1.26% to 4.55%). The superior germplasm identified for curcuminoids content were as follows; curcumin (CL 157 – 2.17% and CL 272 – 2.13%), demethoxycurcumin (CL 253 – 1.45% and CL 157 – 1.31%), bisdemethoxycurcumin (CL 216 – 1.24% and CL 57 – 1.11%) and total curcuminoid content (CL 157 – 4.55% and CL 272 – 4.37%). Clustering based on dendrogram, grouped 200 accessions into seven clusters. Among seven clusters, the maximum number of accessions were grouped into cluster II while cluster VII showed maximum mean value for majority of the traits. Correlation analysis revealed a significant relationship between the traits where the total curcuminoid content is significantly and positively correlated with the primary rhizome core diameter and length of the secondary rhizome. The selection of these particular traits may result in the identification of germplasm with high total curcuminoid content. Taken together, it is the first report on the large screening of turmeric accessions for variation in the rhizome yield traits and curcuminoids content. The genetic diversity revealed in this study could be useful for further crop improvement programs in turmeric to develop new varieties with high rhizome yield coupled with high curcuminoids content.

## Introduction

Turmeric (*Curcuma longa* L.) belongs to the genus *Curcuma* (Zingiberaceae) and, is a commercially important crop because of its pharmacological, nutritional, religious, and cultural significance. The crop is indigenous to India and has a very long history of cultivation in Asia. The genus *Curcuma* contains about 80 species all over Asia. The other important species related to turmeric are *C. amada*, *C. angustifolia*, *C. aromatica*, *C. caesia* and *C. zedoaria* (Ravindran et al., 2007). Turmeric is a cross-pollinated, triploid species (2*n* = 3*x* = 63), which can be vegetatively propagated using its underground rhizomes (Sasikumar, 2005). Curcuminoid compounds (Curcumin, demethoxycurcumin, and bisdemethoxycurcumin) isolated from the rhizomes of turmeric possess various pharmacological activities. In particular, curcumin is reported as a valuable anti-inflammatory, antioxidant and anti-microbial compound (Karthikeyan et al., 2021).

Genetic improvement work on turmeric is typically limited to germplasm selection as hybridization is unsuccessful in many cases. To assess the genetic diversity, genetic relationship among the germplasm is the backbone of any crop improvement program. This can support breeding activities by both farmers and plant breeders. Many studies used the rhizome yield traits to assess the genetic diversity and there are only few studies which characterized the germplasm based on the curcuminoids content. For instance, research findings of Jan et al. (2012) concluded that the evaluated turmeric genotypes based on agro-morphological traits have narrow genetic base and opined that the reason might be due to high level of genetic erosion and selection pressure from farming communities. A research on screening 83 turmeric genotypes for diversity analysis based on morphological characters was conducted by Verma et al. (2014) where high coefficient of variation was observed for number of secondary rhizomes per plant. In an experiment, Singh et al. (2013) screened 60 turmeric genotypes for evaluating curcumin content. Their results revealed the presence of wide variation with respect to curcuminoids content and the value ranged from 0.4% to 8.8% when estimated through Spectrophotometer. In a study, Chao et al. (2018) conducted research on 12 different turmeric genotypes where the curcumin content ranged from 1.09% to 1.64%, demethoxycurcumin content ranged from 0.26% to 0.76% and bisdemethoxycurcumin content ranged from 0.27% to 0.58%. According to Miyazaki et al. (2014) the differences in curcumin yield among the species and strains were mainly due to curcumin content. However, the factor that determines the curcumin yield is diverse in various groups. In the first group (Japanese and Southeast Asian *C. longa* collections), it is due to both rhizome yield and curcumin content, the rhizome yield in second group (Southeast Asian *C. longa* collections) while it is curcumin content in third group (*C. aromatica* and other *Curcuma* species).

Curcumin content in rhizomes may vary depending on the germplasm. Studies focusing on estimating individual curcuminoid content by screening large number of germplasm might help in selecting the germplasm with high individual curcuminoids content. This is required because when the price is fixed based on curcumin content, farmers are willing to grow varieties with high curcumin content. Depending on demand in markets, traders are even willing to buy turmeric rhizomes with high curcumin content (Padmaja and Kiran, 2017). Earlier studies reported that curcuminoids constitutes about 2-4% of dry turmeric root powder (Stohs et al., 2020) and commercially available curcuminoids consists of a mixture of curcumin, demethoxycurcumin and bisdemethoxycurcumin with curcumin as the major constituent (Paramasivam et al., 2009). Curcumin’s low water solubility, poor bioavailabilty, and rapid metabolism restrict for its successful therapeutic applications (Lopresti, 2018; Hatamipour et al., 2019; Stohs et al., 2020). However, demethoxycurcumin and bisdemethoxycurcumin, which lacks methoxy group on the benzene ring of the parent structure (curcumin), has much greater chemical stability at physiological state or acidic pH of human digestive system (Hatamipour et al., 2019). Therefore, identifying the germplasm particularly with high demethoxycurcumin and bisdemethoxycurcumin content could be benefited for both farmers and industries.

India is the centre of turmeric diversity in particular Southern India (i.e., Tamil Nadu, and Kerala). Indian turmeric accessions exhibit good rhizome yield, varying curcuminoids content, and has potential to adapt different agro-climatic zones. As the farmers and industries are not aware of the characteristics of existing germplasm, particularly with regard to total or individual curcuminoid content, they are facing difficulty in recognizing the superior germplasm. With this backdrop, the objective of the present study was to explore the diversity among 200 turmeric accessions for rhizome yield traits and curcuminoids content. In addition, clustering and correlation studies were performed to understand the relationship between the traits.

## Materials and methods

### Germplasm panel

The experimental material consisted of 200 C*. longa* accessions along with two check varieties viz., CO2 (Local check) and CIM PITAMBER (National check). The turmeric accessions were originated from different parts of India (**Supplementary Table 1**). All these plant materials were obtained from turmeric germplasm repository, Department of Spices and Plantation Crops, Horticultural College and Research Institute, Tamil Nadu Agricultural University, Coimbatore, India.

### Experimental site and design

**Figure 1:**
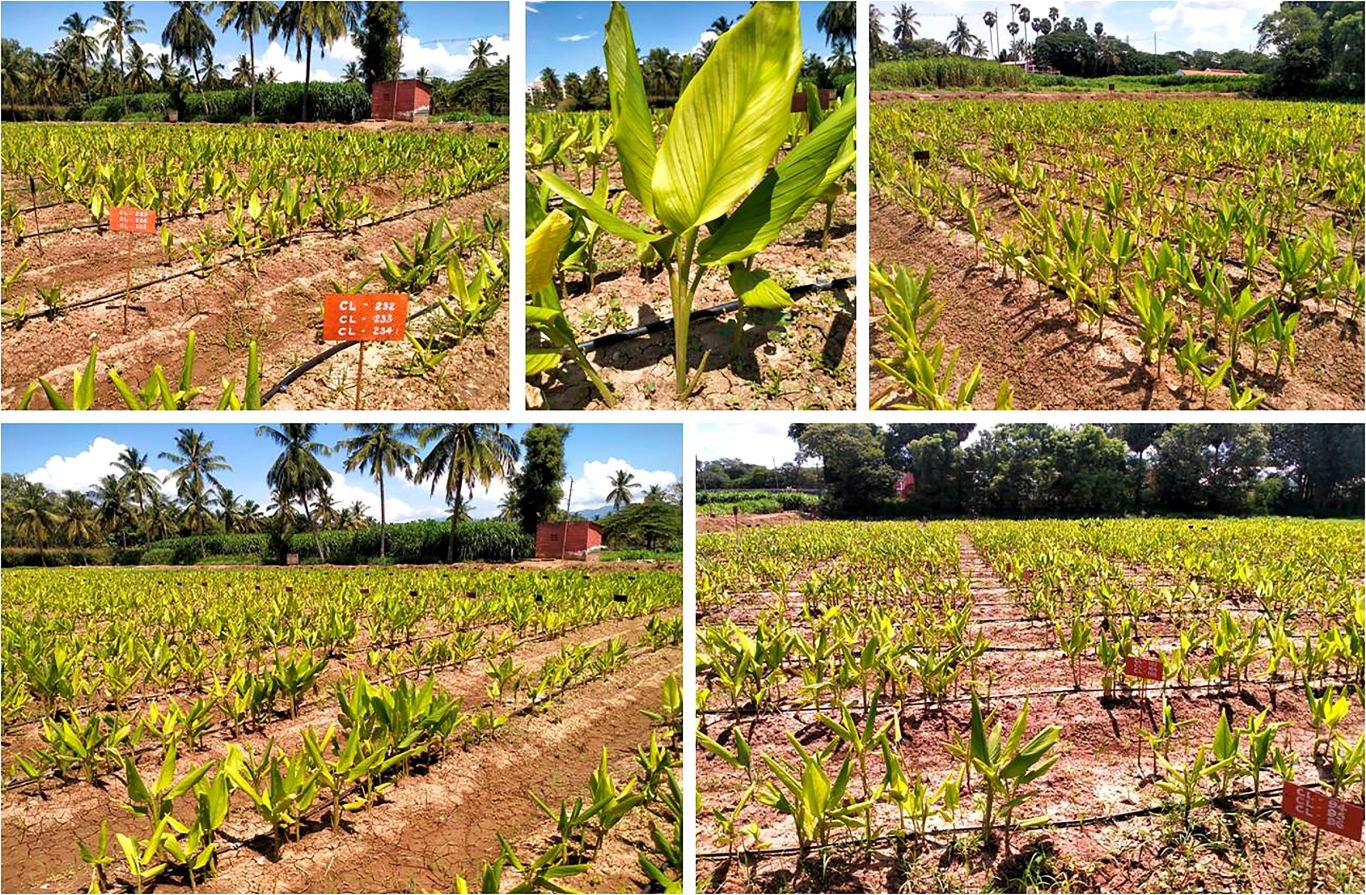
The field view of 200 turmeric accessions at the College Orchard, Horticultural College and Research Institute, Tamil Nadu Agricultural University, Coimbatore.

The experiment was conducted from June 2020 to February 2021 at the College Orchard, Department of Spices and Plantation Crops, Horticultural College and Research Institute, Tamil Nadu Agricultural University, Coimbatore, India. The experimental site was situated at 11° 7’ N latitude, 77° 59’ E longitude, and at an altitude of 426 m above mean sea level (MSL). The experiment was laid out in Augmented Block Design (ABD) with 20 blocks. Each turmeric accession was planted in two rows in a 3 m^2^ plot with a spacing of 45 cm × 15 cm (**Figure 1**). All the recommended practices, including fertilizer application and pest and disease management, were followed during the entire crop period.

### Trait measurement

Rhizomes were harvested when the plant leaves were completely withered. After harvesting, rhizomes were evaluated for 15 traits, *viz.*, fresh rhizome yield (g p^-1^), dry recovery (%), number of mother rhizomes per plant, number of primary rhizomes per plant, number of secondary rhizomes per plant, weight of mother rhizomes (g p^-1^), weight of primary rhizomes (g p^-1^), weight of secondary rhizomes (g p^-1^), length of mother rhizome (cm), girth of mother rhizome (cm), length of primary rhizome (cm), girth of primary rhizome (cm), primary rhizome diameter (cm), primary rhizome core diameter (cm) and length of secondary rhizome (cm) according to the DUS guidelines given by Protection of Plant Varieties and Farmers Rights Authority, Government of India. For dry recovery, one kilogram of fresh rhizomes from each turmeric accession and check varieties were sliced to a thickness of 2 mm. It was further spread on the floor for drying under a glass house until the moisture content was sufficiently reduced to less than 10% (approximately seven days until the sliced rhizomes are brittle enough). Finally, the dry recovery percentage was calculated using the following formula,


$$DR\;\left(\%\right)\;=\;100-\;\left[\left\{W_{1}-W_{2}/W_{1}\right\}\;x\;100\right]$$

Where W_1_ is the fresh weight of the rhizomes and W_2_ is the dry weight of the rhizomes.

### Estimation of total and individual curcuminoid content through HPTLC

Powdered samples of 200 accessions and two check varieties (0.2 g each) were extracted separately in 10 mL of acetone for 12 hours, filtered, and evaporated until near dryness. The individual sample extracts were re-dissolved with 2.0 mL of acetone for quantification. Stock solutions of curcumin (1 mg mL^-1^), demethoxycurcumin (1 mg mL^-1^) and bisdemethoxycurcumin (0.5 mg mL^-1^) were prepared in acetone and were loaded on a TLC plate. Chromatography was performed on a pre-activated (110°C) silica gel TLC plate 60 F_254_ (20 x 10 cm). Standards and samples were applied to the plate as 8 mm wide bands with an automatic TLC applicator, Linomat 5 (CAMAG, Muttenz, Switzerland). The TLC plates were developed using a CAMAG twin trough glass tank (20 X 10 cm), which was pre-saturated with mobile phase chloroform-methanol (95:5) for one hour, and each plate was developed to a height of about 8 cm. TLC was run at 25 ± 5°C and 50% relative humidity. After development, the TLC plates were removed, allowed to dry for a few minutes, and spots were visualized under a UV chamber (CAMAG, Switzerland) at 366 nm. Curcuminoids were quantified with a CAMAG TLC Scanner 4 equipped with Vision CATS software and a computer. The slit width is kept at 6 x 0.45 mm. A wavelength of 436 nm is used with absorption/reflection detection mode.

### Estimation of total and individual curcuminoid content through UHPLC

UHPLC was performed with Schimadzu N series UHPLC system equipped with binary solvent manager, auto sampler (SIL – 40C XS), a quaternary pump (LC 40 DXS), column manager and a PDA detector. The sampling speed maintained at 5 µL sec^-1^. The detector used was SPD-M40. Chromatographic separation was performed on CTO-40S column. The mobile phase used in the study was acetonitrile and 1% glacial acetic acid in isocratic mode, which was degassed before UHPLC analysis. Flow rate of mobile phase was 0.25 mL min^-1^. Sample injection volume was 10 µL. The chromatographic run time per sample was 12 min. The column was maintained at 40°C whereas the auto sampler was maintained at 4°C. Standard curve for curcumin, demethoxycurcumin and bisdemethoxycurcumin was obtained by using standard solutions in the concentration range between 1 µg mL^-1^ to 50 µg mL^-1^ (1, 5, 10, 25 and 50 µg mL^-1^).

### Statistical analysis

All the data of traits were statistically analyzed. Variability and diversity were computed as per the methods suggested by Johnson et al. (1955) and Rao (1952). Heat map, dendrogram, and PCA were constructed by using R-Software. Correlation between all the observed traits was studied by applying the Pearson correlation coefficient using SPSS 20.0 Version and R –Software.

## Results

### Performance of turmeric germplasm based on the rhizome yield traits

**Figure 2:**
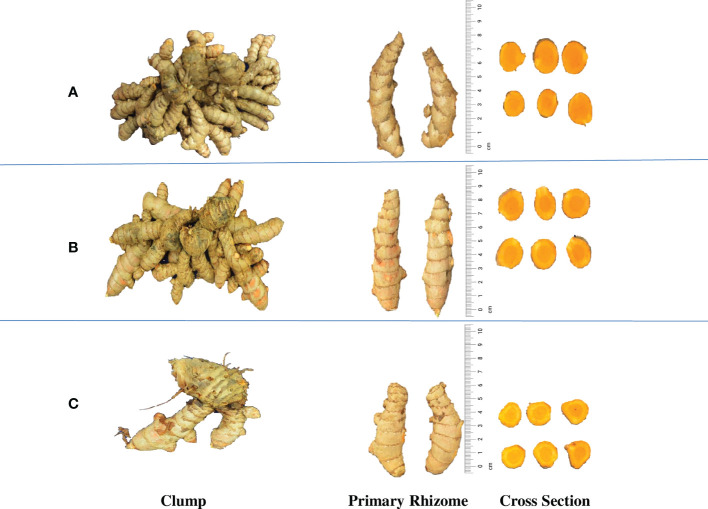
Rhizome yield variation in the turmeric accessions. **(A)** CL 180, High yielding accession **(667.63** g **p^-1^)**, **(B)** CL 157, Medium yielding accession **(359.32** g **p^-1^)** and **(C)** CL 5, Low yielding accession **(24.77** g **p^-1^)**.

Turmeric accessions exhibited large variation for the following traits viz., weight of secondary rhizomes per plant (50.23%), weight of mother rhizomes per plant (46.77%), weight of primary rhizomes per plant (38.83%), number of primary rhizomes per plant (38.29%), number of secondary rhizomes per plant (37.51%), number of mother rhizomes per plant (36.69%) and yield per plant (33.43%) (**Supplementary Table 2**). The mean, range, coefficient of variation and critical difference (CD) value for the traits were represented in **Table 1**. The number of turmeric accessions that are significantly superior over the mean of a particular trait was as follows. The number of primary rhizomes per plant (79 germplasm accessions), yield (64 germplasm accessions), dry recovery (53 germplasm accessions), number of secondary rhizomes per plant (39 germplasm accessions), weight of secondary rhizomes per plant (35 germplasm accessions), weight of primary rhizomes per plant (32 germplasm accessions), weight of mother rhizomes per plant (29 germplasm accessions), number of mother rhizomes per plant (21 germplasm accessions), length of secondary rhizome (16 germplasm accessions) and length of mother rhizome (15 germplasm accessions). The following traits recorded significantly less number of turmeric accessions over the mean value, i.e., length of primary rhizome (one germplasm accession), primary rhizome diameter (two germplasm accessions), primary rhizome core diameter (five germplasm accessions) and girth of mother rhizome (seven germplasm accessions). In contrast, the girth of the primary rhizome doesn’t have any significant accessions over the mean value. The yield of germplasm ranged from 24.77 to 667.63 g p^-1^ with an average of 357.74 g p^-1^. The maximum yielding accession was CL 180, whereas the minimum yielder was CL 5 (**Figure 2**). The dry recovery percentage of the germplasm lies between 13% and 29%, where the maximum value was shown by CL 9, and the minimum was found with CL 220. The average dry recovery percentage of the germplasm was 20%. The weight of mother rhizomes per plant ranged from 8.17 g (CL 5) to 229.28 g (CL 161), with an average of 91.48 g. The weight of primary rhizomes per plant ranged from 12.25 g to 405.93 g, where the minimum and maximum values were obtained by CL 5 and CL 242, respectively. The average weight of primary rhizomes per plant recorded was 189.72 g. The weight of secondary rhizomes per plant ranged from 4.35 g to 227.53 g, with an average value of 76.54 g.

**Table 1:** Descriptive statistical analysis for rhizome yield traits and curcuminoids content among 200 turmeric germplasm accessions.

Trait	Mean	Range	CV (%)	CD (P=0.05)
Yield (g p^-1^)	357.74 ± 18.48	24.77 – 667.63	33.43	64.03
DR (%)	20.00 ± 0.85	13.42 – 29.18	19.25	2.96
MRN	3.54 ± 0.64	1.50 – 7.70	36.69	2.25
PRN	12.90 ± 0.27	1.90 – 24.60	38.29	0.95
SRN	13.54 ± 1.45	3.30 – 30.60	37.51	5.05
MRW (g p^-1^)	91.48 ± 14.72	8.17 – 229.28	46.77	51.02
PRW (g p^-1^)	189.72 ± 20.71	12.25 – 405.93	38.83	71.77
SRW (g p^-1^)	76.54 ± 10.03	4.35 – 227.53	50.23	34.76
MRL (cm)	5.07 ± 0.29	3.30 – 7.90	14.18	1.02
MRG (cm)	8.50 ± 0.55	6.10 – 12.20	12.25	1.91
PRL (cm)	7.96 ± 0.64	5.20 – 10.30	11.31	2.23
PRG (cm)	6.51 ± 0.57	4.70 – 8.00	10.3	1.99
PRD (cm)	2.11 ± 0.21	1.10 – 2.90	15.78	0.73
PRCD (cm)	1.19 ± 0.14	0.40 – 2.00	24.65	0.51
SRL (cm)	3.81 ± 0.30	2.50 – 6.00	18.19	1.05
A (%)	1.38 ± 0.01	0.41 – 2.17	24.31	0.06
B (%)	0.89 ± 0.02	0.38 – 1.45	19.75	0.08
C (%)	0.74 ± 0.03	0.37 – 1.24	18.77	0.12
TCC (%)	3.01 ± 0.02	1.26 – 4.55	19.69	0.08

(DR) Dry Recovery, (MRN) Number of Mother Rhizomes per Plant, (PRN) Number of Primary Rhizomes per Plant, (SRN) Number of Secondary Rhizomes per Plant, (MRW) Weight of Mother Rhizomes per Plant, (PRW) Weight of Primary Rhizomes per Plant, (SRW) Weight of Secondary Rhizomes per Plant, (MRL) Length of Mother Rhizome, (MRG) Girth of Mother Rhizome, (PRL) Length of Primary Rhizome, (PRG) Girth of Primary Rhizome, (PRD) Primary Rhizome Diameter, (PRCD) Primary Rhizome Core Diameter, (SRL) Length of Secondary Rhizome, (A) Curcumin, (B) Demethoxycurcumin, (C) Bisdemethoxycurcumin, and (TCC) Total Curcuminoid Content.

### Performance of turmeric germplasm based on curcuminoids content

**Figure 3:**
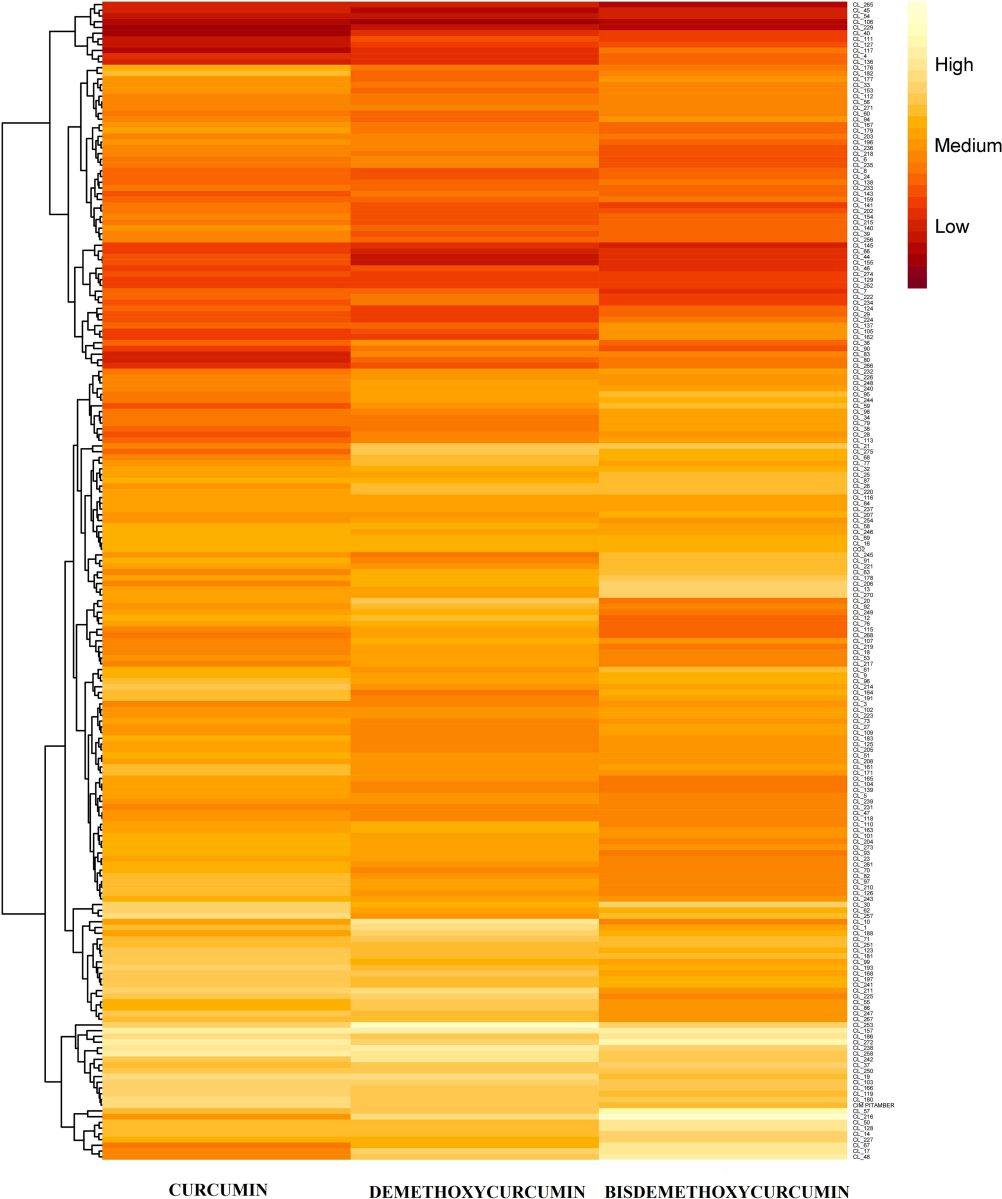
Heat-map chart of curcuminoids content among turmeric accessions along with check varieties.

**Figure 4:**
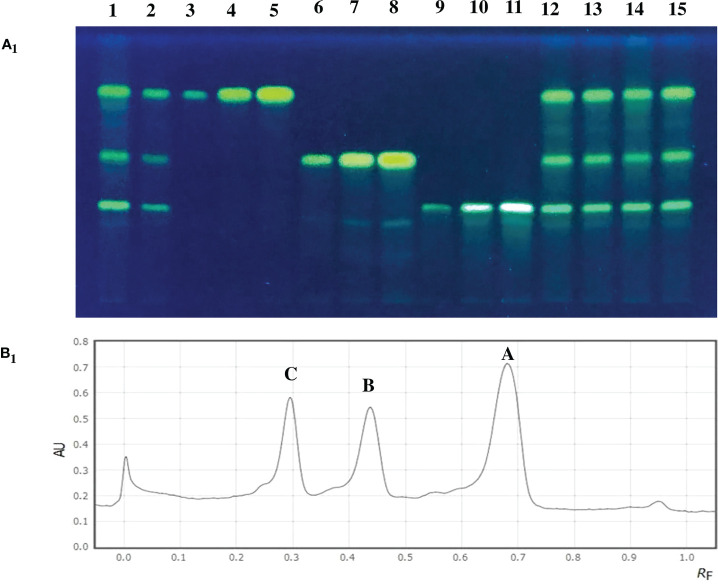
**(A_1_)** TLC plate showing curcuminoid fractions of turmeric accessions along with standard compounds at 366 nm. (Lane 1) CL-94, (Lane 2) CL-252, (Lane 3-5) Standard curcumin (1 µl, 3 µl and 6 µl), (Lane 6-8) Standard demethoxycurcumin (1 µl, 3 µl and 6 µl), (Lane 9-11) Standard bisdemethoxycurcumin (1 µl, 3 µl and 6 µl), (Lane 12) CL-112, (Lane 13) CL-215, (Lane 14) CL-224 and (Lane 15) CL-231. **(B_1_)** HPTLC separation of curcuminoid fractions from the check variety CO 2. (A) Curcumin (1.58%), (B) Demethoxycurcumin (0.97%), (C) Bisdemethoxycurcumin (0.82%). (AU) Absorbption intensity and (R_F_) Retention factor.

Germplasm exhibited wide variation for total curcuminoid content (**Supplementary Table 3** and **Figure 3**). The number of germplasm accessions that are significantly superior over the mean of curcumin content was 91, whereas, for demethoxycurcumin content, it is 60 germplasm accessions, 31 germplasm accessions for bisdemethoxycurcumin content, and 100 germplasm accessions for total curcuminoid content. HPTLC quantification of total curcuminoid content and their fractions (**Figure 4**) revealed that, curcumin content ranged from 0.41% (CL 40) to 2.17% (CL 157), demethoxycurcumin ranged from 0.38% (CL 106) to 1.45% (CL 253) and bisdemethoxycurcumin ranged from 0.37% (CL 229) to 1.24% (CL 216) whereas, total curcuminoid content ranged from 1.26% (CL 229) to 4.55% (CL 157). The average total curcuminoid content was 3.01%. As a part of confirmation studies, we have selected 15 accessions from three groups (five accessions each from high, medium and low total curcuminoid containing germplasm) and analyzed total and individual curcuminoid content through UHPLC. The total and individual curcuminoid content of the selected accessions were given in **Table 2**. The minimum and maximum curcuminoids content among 15 selected accessions were as follows, curcumin (0.31% and 1.73%); demethoxycurcumin (0.25% and 0.99%) and bisdemethoxycurcumin (0.26% and 0.75%). The range of total curcuminoid content was between 0.93% to 3.38% with a mean of 2.17%. It was observed that, the values among three groups were on same trend between two methods (UHPLC and HPTLC) (**Table 2**).

**Table 2:** Confirmative analysis of total and individual curcuminoid content from 15 selected germplasm accessions through UHPLC.

S. No.	Germplasm Code No.		UHPLC				HPTLC			
			A (%)	B (%)	C (%)	TCC (%)	A (%)	B (%)	C (%)	TCC (%)
**High total curcuminoid containing accessions**										
1	CL 157	1.72		0.91	0.75	3.38	2.17	1.31	1.07	4.55
2	CL 272	1.73		0.76	0.73	3.22	2.13	1.13	1.11	4.37
3	CL 238	1.68		0.90	0.68	3.26	2.08	1.28	0.94	4.30
4	CL 258	1.69		0.84	0.65	3.18	2.10	1.24	0.89	4.23
5	CL 253	1.46		0.99	0.69	3.14	1.81	1.45	0.96	4.22
**Mean**		**1.66**		**0.88**	**0.70**	**3.23**	**2.06**	**1.28**	**0.99**	**4.33**
**Medium total curcuminoid containing accessions**										
6	CL 240	1.02		0.68	0.52	2.21	1.27	0.95	0.8	3.02
7	CL 27	1.19		0.56	0.54	2.29	1.40	0.83	0.78	3.01
8	CL 73	1.15		0.57	0.53	2.25	1.43	0.83	0.75	3.01
9	CL 223	1.08		0.62	0.55	2.25	1.35	0.87	0.78	3.00
10	CL 53	1.07		0.66	0.49	2.22	1.34	0.94	0.7	2.98
**Mean**		**1.10**		**0.62**	**0.52**	**2.24**	**1.36**	**0.88**	**0.76**	**3.00**
**Low total curcuminoid containing accessions**										
11	CL 229	0.32		0.35	0.26	0.93	0.41	0.48	0.37	1.26
12	CL 106	0.40		0.25	0.26	0.91	0.53	0.38	0.40	1.31
13	CL 54	0.45		0.33	0.30	1.08	0.59	0.47	0.45	1.51
14	CL 40	0.31		0.41	0.37	1.09	0.41	0.61	0.56	1.58
15	CL 45	0.52		0.30	0.30	1.12	0.68	0.46	0.45	1.59
**Mean**		**0.40**		**0.33**	**0.30**	**1.02**	**0.52**	**0.48**	**0.45**	**1.45**
**Grand Mean**		**1.05**		**0.61**	**0.51**	**2.17**	**1.31**	**0.88**	**0.73**	**2.93**

**(A)** Curcumin, **(B)** Bisdemethoxycurcumin, **(C)** Bisdemethoxycurcumin, and **(TCC)** Total Curcuminoid Content.

### Germplasm clusters

#### Cluster analysis based on the dendrogram

**Figure 5:**
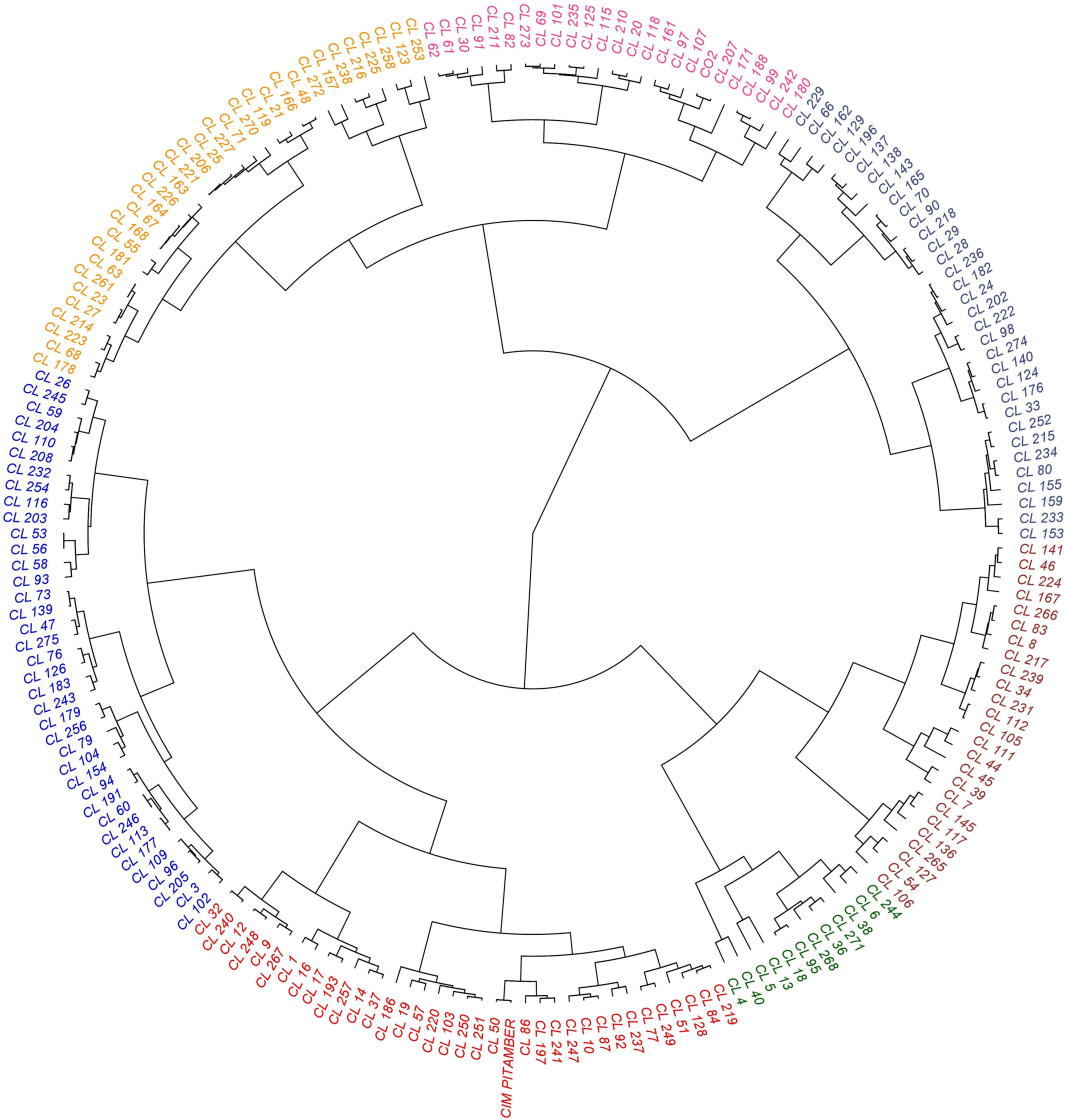
Dendrogram depicting grouping of turmeric accessions based on the rhizome yield traits and curcuminoids content.

All the 200 C*. longa* accessions, including two check varieties, were grouped into seven clusters (**Figure 5**). Among the seven clusters, the highest number of accessions were grouped in cluster II (38 germplasm accessions), followed by cluster III (35 germplasm accessions). The other clusters, such as clusters I, IV, V, VI, and VII, possessed 33, 12, 25, 33 and 24 germplasm accessions, respectively. Check varieties CIM PITAMBER and CO 2 were grouped into cluster III and cluster VII, respectively. Cluster mean for all the characters had considerable differences between the clusters in our study (**Table 3**). Cluster VII showed maximum mean value for most of the traits, viz., yield (516.85 g p^-1^), dry recovery (21.58%), number of mother rhizomes per plant (4.65), number of primary rhizomes per plant (16.48), number of secondary rhizomes per plant (19.42), weight of mother rhizomes (133.17 g p^-1^), weight of primary rhizomes (257.65 g p^-1^), weight of secondary rhizomes (126.03 g p^-1^), length of mother rhizome (5.59 cm), girth of mother rhizome (9.28 cm) and length of primary rhizome (8.50 cm). Cluster I showed maximum mean value for the girth of primary rhizome (6.96 cm), primary rhizome diameter (2.30 cm), primary rhizome core diameter (1.41 cm), length of secondary rhizome (4.19 cm), bisdemethoxycurcumin content (0.88%) and total curcuminoid content (3.52%). Among the clusters, the maximum mean value for demethoxycurcumin content was 1.04%, and the value was the same for both cluster I and cluster III, whereas the maximum mean value for curcumin content (1.61%) was obtained in cluster III.

**Table 3:** Cluster means of 200 turmeric germplasm accessions.

Trait	Cluster I	Cluster II	Cluster III	Cluster IV	Cluster V	Cluster VI	Cluster VII
Yield (g p^-1^)	413.05	334.32	243.22	139.74	318.41	442.86	516.85
DR (%)	19.59	19.92	18.42	19.43	20.76	20.68	21.58
MRN	3.55	3.39	2.64	2.4	3.31	4.38	4.65
PRN	14.33	11.77	9.14	7.42	14.18	15.2	16.48
SRN	15.44	13.01	11.13	8.14	10.84	14.38	19.42
MRW (g p^-1^)	96.61	90.88	59.34	35.37	80.29	119.4	133.17
PRW (g p^-1^)	227.43	171.91	132.94	76.36	171.79	237.79	257.65
SRW (g p^-1^)	89.02	71.52	50.94	28	66.33	85.68	126.03
MRL (cm)	5.22	5.02	4.97	4.33	4.79	5.2	5.59
MRG (cm)	8.75	8.21	8.19	7.43	8.01	9.06	9.28
PRL (cm)	8.44	7.85	7.76	6.8	7.55	8.13	8.5
PRG (cm)	6.96	6.36	6.51	5.65	6.12	6.68	6.7
PRD (cm)	2.3	2.06	2.19	1.68	1.89	2.13	2.23
PRCD (cm)	1.41	1.13	1.25	0.78	0.99	1.2	1.27
SRL (cm)	4.19	3.77	3.78	3.59	3.59	3.73	3.85
A (%)	1.6	1.41	1.61	1.13	0.95	1.13	1.59
B (%)	1.04	0.87	1.04	0.85	0.67	0.73	0.96
C (%)	0.88	0.74	0.84	0.71	0.59	0.63	0.77
TCC (%)	3.52	3.02	3.49	2.69	2.21	2.48	3.32

(DR) Dry Recovery, (MRN) Number of Mother Rhizomes per Plant, (PRN) Number of Primary Rhizomes per Plant, (SRN) Number of Secondary Rhizomes per Plant, (MRW) Weight of Mother Rhizomes per Plant, (PRW) Weight of Primary Rhizomes per Plant, (SRW) Weight of Secondary Rhizomes per Plant, (MRL) Length of Mother Rhizome, (MRG) Girth of Mother Rhizome, (PRL) Length of Primary Rhizome, (PRG) Girth of Primary Rhizome, (PRD) Primary Rhizome Diameter, (PRCD) Primary Rhizome Core Diameter, (SRL) Length of Secondary Rhizome, (A) Curcumin, (B) Demethoxycurcumin, (C) Bisdemethoxycurcumin, and (TCC) Total Curcuminoid Content.

#### Principal component analysis

**Figure 6:**
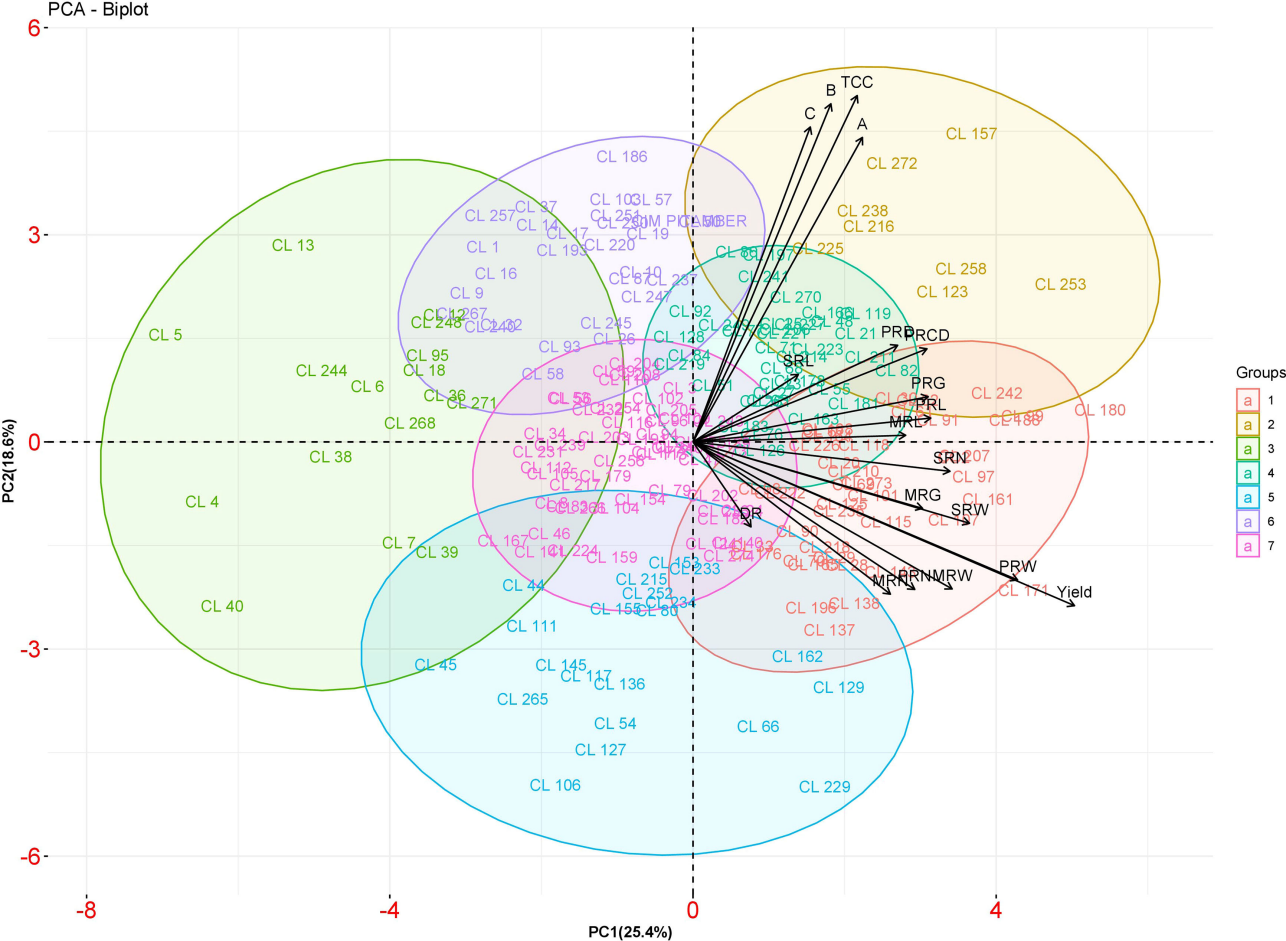
PCA biplot between PC 1 and PC 2 scores of turmeric accessions. The accessions belonging to different groups are indicated by different colors.

The first six principal components accounted for about 78.88% of the total variance (**Table 4**). In the total variance, PC1 accounted for 25.38%, and all the traits were positively contributed wherein yield and weight of primary rhizome showed high contribution. PC2 accounted for 18.60% of the total variance. The highly contributed factor loadings towards PC2 were total curcuminoid content, demethoxycurcumin, bisdemethoxycurcumin, and curcumin. PC3, PC4, PC5, and PC6 contributed 13.23, 9.15, 6.70, and 5.79% of total variance, respectively. Further, a biplot was drawn between PC1 and PC2 to observe the relationship between these two components (**Figure 6**). Turmeric accession CL 180 is a high yielder placed at the extreme right corner of the biplot, whereas CL 5 is a low yielding accession placed at the extreme left corner of the biplot. The contrasting characters were placed in an opposite position.

**Table 4:** Factor loadings of rhizome yield traits and curcuminoids content for the first six principal components.

Trait	PC1	PC2	PC3	PC4	PC5	PC6
Yield (g p^-1^)	0.39	-0.21	-0.13	0.01	0.15	-0.05
DR (%)	0.06	-0.11	-0.17	-0.01	-0.47	0.58
MRN	0.20	-0.20	-0.12	0.44	-0.19	-0.37
PRN	0.23	-0.19	-0.28	0.01	0.41	0.23
SRN	0.26	-0.04	-0.08	-0.44	-0.06	-0.22
MRW (g p^-1^)	0.26	-0.19	-0.09	0.40	-0.21	-0.33
PRW (g p^-1^)	0.33	-0.18	-0.11	0.02	0.38	0.24
SRW (g p^-1^)	0.28	-0.11	-0.09	-0.46	-0.01	-0.24
MRL (cm)	0.22	0.01	0.22	-0.01	-0.35	0.04
MRG (cm)	0.23	-0.09	-0.13	0.06	-0.37	0.31
PRL (cm)	0.24	0.03	0.30	-0.13	0.04	0.17
PRG (cm)	0.24	0.06	0.31	0.02	0.01	0.14
PRD (cm)	0.21	0.13	0.47	0.14	0.05	-0.01
PRCD (cm)	0.24	0.12	0.45	0.11	0.11	0.03
SRL (cm)	0.11	0.09	0.01	-0.41	-0.28	-0.21
A (%)	0.17	0.40	-0.22	0.09	-0.02	-0.01
B (%)	0.14	0.44	-0.18	0.05	0.00	0.05
C (%)	0.12	0.41	-0.16	0.06	0.06	-0.04
TCC (%)	0.17	0.45	-0.21	0.08	0.00	0.00
Eigen value	4.82	3.53	2.51	1.73	1.27	1.10
Percent variance	25.38	18.60	13.23	9.15	6.70	5.79
Cumulative variance	25.38	43.98	57.22	66.38	73.08	78.88

(DR) Dry Recovery, (MRN) Number of Mother Rhizomes per Plant, (PRN) Number of Primary Rhizomes per Plant, (SRN) Number of Secondary Rhizomes per Plant, (MRW) Weight of Mother Rhizomes per Plant, (PRW) Weight of Primary Rhizomes per Plant, (SRW) Weight of Secondary Rhizomes per Plant, (MRL) Length of Mother Rhizome, (MRG) Girth of Mother Rhizome, (PRL) Length of Primary Rhizome, (PRG) Girth of Primary Rhizome, (PRD) Primary Rhizome Diameter, (PRCD) Primary Rhizome Core Diameter, (SRL) Length of Secondary Rhizome, (A) Curcumin, (B) Demethoxycurcumin, (C) Bisdemethoxycurcumin, and (TCC) Total Curcuminoid Content.

### Contribution of each trait to total divergence

**Figure 7:**
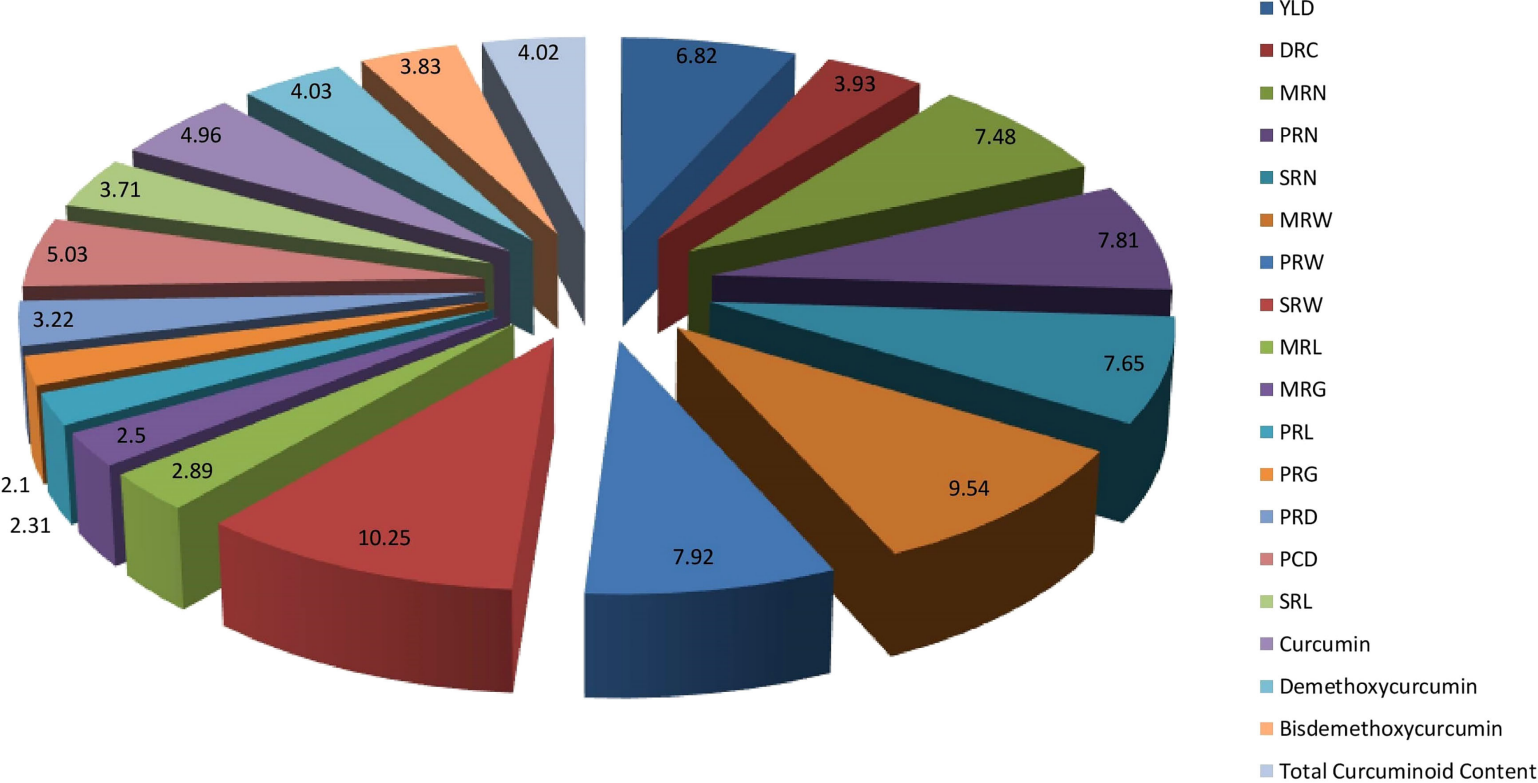
Percent contribution of rhizome yield traits and curcuminoids content to total divergence. (DR) Dry Recovery, (MRN) Number of Mother Rhizomes per Plant, (PRN) Number of Primary Rhizomes per Plant, (SRN) Number of Secondary Rhizomes per Plant, (MRW) Weight of Mother Rhizomes per Plant, (PRW) Weight of Primary Rhizomes per Plant, (SRW) Weight of Secondary Rhizomes per Plant, (MRL) Length of Mother Rhizome, (MRG) Girth of Mother Rhizome, (PRL) Length of Primary Rhizome, (PRG) Girth of Primary Rhizome, (PRD) Primary Rhizome Diameter, (PRCD) Primary Rhizome Core Diameter, and (SRL) Length of Secondary Rhizome.

The per cent contribution of each character to the total divergence was obtained by ranking the individual trait. The trait, weight of secondary rhizomes per plant contributed the maximum to the divergence, i.e., 10.25%, followed by weight of mother rhizomes per plant (9.54%) and weight of primary rhizomes per plant (7.92%) (**Figure 7**).

### Pearson correlation analysis

**Figure 8:**
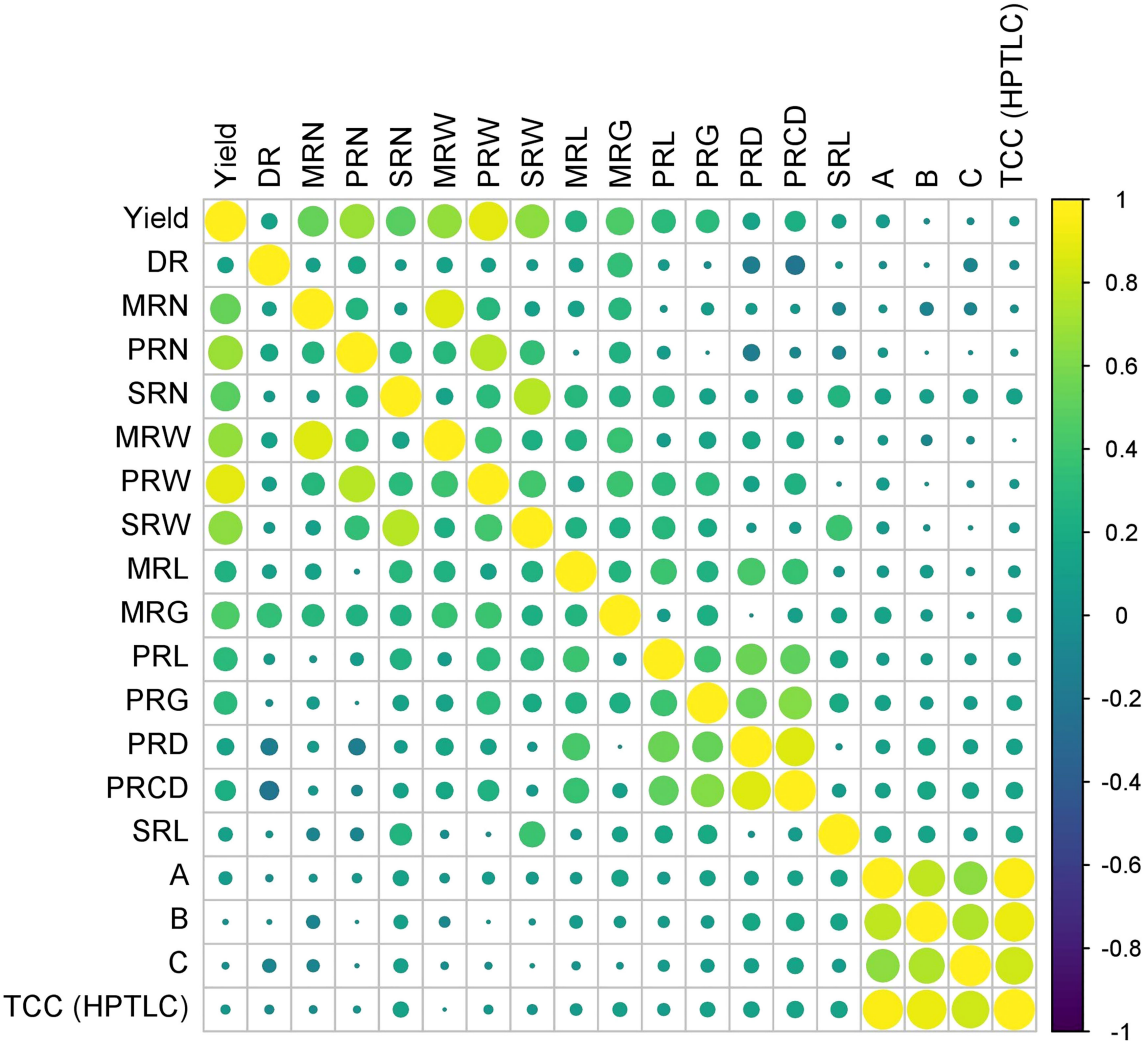
Pearson correlation coefficients between rhizome yield traits and curcuminoids content in turmeric germplasm. (DR) Dry Recovery, (MRN) Number of Mother Rhizomes per Plant, (PRN) Number of Primary Rhizomes per Plant, (SRN) Number of Secondary Rhizomes per Plant, (MRW) Weight of Mother Rhizomes per Plant, (PRW) Weight of Primary Rhizomes per Plant, (SRW) Weight of Secondary Rhizomes per Plant, (MRL) Length of Mother Rhizome, (MRG) Girth of Mother Rhizome, (PRL) Length of Primary Rhizome, (PRG) Girth of Primary Rhizome, (PRD) Primary Rhizome Diameter, (PRCD) Primary Rhizome Core Diameter, (SRL) Length of Secondary Rhizome, (A) Curcumin, (B) Demethoxycurcumin, (C) Bisdemethoxycurcumin, and (TCC) Total Curcuminoid Content.

Pearson correlation analysis was performed between 19 characters of turmeric germplasm, and correlation coefficients were given in **Table 5** and **Figure 8**. The rhizome yield per plant exhibited a highly significant positive correlation with the following characters viz., weight of primary rhizomes per plant (0.890), number of primary rhizomes per plant (0.690), weight of mother rhizomes per plant (0.672), weight of secondary rhizomes per plant (0.658), number of mother rhizomes per plant (0.532), number of secondary rhizomes per plant (0.498), girth of mother rhizome (0.444), length of primary rhizome (0.320), girth of primary rhizome (0.304), length of mother rhizome (0.254) and primary rhizome core diameter (0.234). The rhizome yield per plant exhibited a significant positive correlation with primary rhizome diameter (0.157). In case of dry recovery %, it exhibited a highly significant positive correlation with the girth of the mother rhizome (0.346) and a significant positive correlation with the number of primary rhizomes per plant (0.162), whereas the dry recovery percentage exhibited a highly significant negative correlation with primary rhizome core diameter (-0.203) and significant negative correlation with primary rhizome diameter (- 0.158). The rest of the traits were non-significant with dry recovery percentage. Total curcuminoid content is highly significant and positively correlated with curcumin (0.948), demethoxycurcumin (0.915), and bisdemethoxycurcumin (0.821), whereas the total curcuminoid content had a significant positive correlation with primary rhizome core diameter (0.155) and length of secondary rhizome (0.152). The rest of the traits were non-significant with total curcuminoid content.

**Table 5:** Pearson correlation coefficients between the traits of turmeric accessions.

	Yield (g p^-1^)	DR(%)	MRN	PRN	SRN	MRW (g p^-1^)	PRW (g p^-1^)	SRW (g p^-1^)	MRL(cm)	MRG(cm)	PRL(cm)	PRG(cm)	PRD(cm)	PRCD(cm)	SRL(cm)	A(%)	B(%)	C(%)	TCC(%)
Yield	1	.136	.532^**^	.690^**^	.498^**^	.672^**^	.890^**^	.658^**^	.254^**^	.444^**^	.320^**^	.304^**^	.157^*^	.234^**^	.104	.092	-.013	-.022	.043
DR	.136	1	.106	.162^*^	.062	.132	.111	.063	.107	.346^**^	.060	-.022	-.158^*^	-.203^**^	.022	-.026	-.010	-.099	-.041
MRN	.532^**^	.106	1	.270^**^	.080	.873^**^	.296^**^	.117	.137	.282^**^	.023	.082	.065	.044	-.089	.031	-.096	-.082	-.030
PRN	.690^**^	.162^*^	.270^**^	1	.262^**^	.290^**^	.774^**^	.341^**^	-.011	.256^**^	.091	.001	-.153^*^	-.061	-.095	.046	.001	-.007	.025
SRN	.498^**^	.062	.080	.262^**^	1	.151^*^	.314^**^	.780^**^	.291^**^	.247^**^	.258^**^	.138	.088	.121	.273^**^	.132	.105	.111	.131
MRW	.672^**^	.132	.873^**^	.290^**^	.151^*^	1	.393^**^	.224^**^	.252^**^	.365^**^	.098	.155^*^	.167^*^	.160^*^	-.035	.048	-.062	-.028	.002
PRW	.890^**^	.111	.296^**^	.774^**^	.314^**^	.393^**^	1	.416^**^	.136	.385^**^	.308^**^	.307^**^	.134	.252^**^	-.009	.081	.005	-.024	.042
SRW	.658^**^	.063	.117	.341^**^	.780^**^	.224^**^	.416^**^	1	.248^**^	.236^**^	.294^**^	.184^**^	.045	.066	.381^**^	.079	.018	.008	.052
MRL	.254^**^	.107	.137	-.011	.291^**^	.252^**^	.136	.248^**^	1	.267^**^	.383^**^	.249^**^	.429^**^	.373^**^	.059	.071	.089	.035	.075
MRG	.444^**^	.346^**^	.282^**^	.256^**^	.247^**^	.365^**^	.385^**^	.236^**^	.267^**^	1	.084	.235^**^	.000	.115	.127	.148^*^	.071	.021	.110
PRL	.320^**^	.060	.023	.091	.258^**^	.098	.308^**^	.294^**^	.383^**^	.084	1	.396^**^	.551^**^	.501^**^	.168^*^	.080	.068	.068	.082
PRG	.304^**^	-.022	.082	.001	.138	.155^*^	.307^**^	.184^**^	.249^**^	.235^**^	.396^**^	1	.533^**^	.625^**^	.196^**^	.130	.087	.095	.121
PRD	.157^*^	-.158^*^	.065	-.153^*^	.088	.167^*^	.134	.045	.429^**^	.000	.551^**^	.533^**^	1	.878^**^	.017	.106	.164^*^	.115	.135
PRCD	.234^**^	-.203^**^	.044	-.061	.121	.160^*^	.252^**^	.066	.373^**^	.115	.501^**^	.625^**^	.878^**^	1	.098	.121	.177^*^	.144^*^	.155^*^
SRL	.104	.022	-.089	-.095	.273^**^	-.035	-.009	.381^**^	.059	.127	.168^*^	.196^**^	.017	.098	1	.148^*^	.154^*^	.096	.152^*^
A	.092	-.026	.031	.046	.132	.048	.081	.079	.071	.148^*^	.080	.130	.106	.121	.148^*^	1	.783^**^	.642^**^	.948^**^
B	-.013	-.010	-.096	.001	.105	-.062	.005	.018	.089	.071	.068	.087	.164^*^	.177^*^	.154^*^	.783^**^	1	.751^**^	.915^**^
C	-.022	-.099	-.082	-.007	.111	-.028	-.024	.008	.035	.021	.068	.095	.115	.144^*^	.096	.642^**^	.751^**^	1	.821^**^
TCC	.043	-.041	-.030	.025	.131	.002	.042	.052	.075	.110	.082	.121	.135	.155^*^	.152^*^	.948^**^	.915^**^	.821^**^	1

(DR) Dry Recovery, (MRN) Number of Mother Rhizomes per Plant, (PRN) Number of Primary Rhizomes per Plant, (SRN) Number of Secondary Rhizomes per Plant, (MRW) Weight of Mother Rhizomes per Plant, (PRW) Weight of Primary Rhizomes per Plant, (SRW) Weight of Secondary Rhizomes per Plant, (MRL) Length of Mother Rhizome, (MRG) Girth of Mother Rhizome, (PRL) Length of Primary Rhizome, (PRG) Girth of Primary Rhizome, (PRD) Primary Rhizome Diameter, (PRCD) Primary Rhizome Core Diameter, (SRL) Length of Secondary Rhizome, (A) Curcumin, (B) Demethoxycurcumin, (C) Bisdemethoxycurcumin, and (TCC) Total Curcuminoid Content. *Significant; **Highly Significant.

## Discussion

The evaluation of genetic variation can provide information about the uniqueness and distinctness of germplasm accessions, which is very important in the effective conservation and utilization of particular germplasm accession (Bisrat et al., 2000 and Dev et al., 2017). Descriptive statistical analysis of rhizome yield traits and curcuminoids revealed the presence of large variation among germplasm. Similar results were obtained by Aarthi et al. (2018) and Prasath and Sasikumar (2016). Among 200 turmeric accessions, CL 180 recorded the maximum yield (667.63 g p^-1^), but the dry recovery percentage of CL 180 was significantly low when compared with the accession having maximum dry recovery (29.18%). But, the accession with maximum dry recovery percentage (CL 9) is a low yielder (119.23 g p^-1^). CL 161 is one of the top three high-yielding accessions and also possessed values that are significantly superior over the means of dry recovery percentage, number of mother rhizomes per plant, and the weight of primary rhizomes per plant. In addition, this CL 161 was found to have the maximum weight of mother rhizomes per plant (229.28 g p^-1^). Similar research on 19 turmeric genotypes at high altitude region of Andhra Pradesh was executed by Krishna et al. (2019). Their results indicated that, CLA 3 recorded maximum fresh rhizome yield per plant (658.32 g), followed by CLA 5 (574.352 g) whereas dry rhizome yield per plant was maximum in CLA 5 (123.14 g) followed by CLA 3 (114.94 g).

The germplasm exhibited wide variation for curcuminoids content viz., curcumin (0.41% to 2.17%), demethoxycurcumin (0.38% to 1.45%), bisdemethoxycurcumin (0.37% to 1.24%) and total curcuminoid content (1.26% to 4.55%). In an experiment, Pathania et al. (2006) conducted a study by collecting turmeric rhizome samples from nine different locations in the western Himalayas. They concluded that curcumin content ranged from 1.26% to 3.76%, demethoxycurcumin content ranged from 0.52% to 2.53% and bisdemethoxycurcumin content ranged from 0.57% to 0.64% while the total curcuminoid content lies between 2.55% to 7.91%. The range of the results reported by Pathania et al. (2006) slightly varied with the result of the present study. The reason might be due to the geographical location where those plant material were collected from a higher latitude (31° 59’ N) whereas the present study was conducted at a lower latitude (11° 7’ N). However, the ratio of curcuminoid fractions was similar with the results of the present study. According to Demasi et al. (2018), latitude gradient effected the secondary metabolite production in *Lavandula angustifolia*. Similar outcomes were also attained by Paramasivam et al. (2009), where the total curcuminoid content ranged from 1.65% to 6.18%. In our study, among the three curcuminoids, curcumin content was highest in most of the turmeric accessions, followed by demethoxycurcumin and bisdemethoxycurcumin content. Earlier studies conducted by Pathania et al. (2006) and Chao et al. (2018) also found similar results. UHPLC analysis of 15 selected accessions recorded the concentration of three curcuminoids in the order of curcumin > demethoxycurcumin > bisdemethoxycurcumin in most of the samples except CL 40. Same trend was observed even in the HPTLC result. The range of UHPLC values obtained in the study was in accordance with previous work carried out by Ashraf et al. (2015). The grand mean of 15 selected accessions for total curcuminoid content was 2.17% when analyzed through UHPLC, whereas it was 2.93% when analyzed through HPTLC. When the HPTLC result was compared to UHPLC result, there is a reduction in the values of UHPLC. The reason for lower values in UHPLC might be due to its ultra-high sensitivity whereas it is just moderate in HPTLC (Jain et al., 2014). However, it was observed that, the values among three groups were on same trend between the two methods (UHPLC and HPTLC).

To explore the diversity among 200 C*. longa* accessions, the data on both rhizome yield traits and curcuminoids content were analyzed using cluster analysis and PCA. The distribution pattern of accessions among the clusters reflected the significant variability in the germplasm. Clustering based on both rhizome yield traits and curcuminoids content has divided the germplasm into seven clusters. Among the seven clusters, cluster VII registered maximum mean value for fresh rhizome yield (516.85 g p^-1^) whereas cluster I recorded maximum mean value for total curcuminoid content (3.52%). Interestingly, cluster VII which recorded maximum mean for fresh rhizome yield was also having third highest mean value for total curcuminoid content (3.32%). Similar trend was observed in cluster I which recorded maximum total curcuminoid content was also found to have third highest mean for fresh rhizome yield (413.05 g p^-1^). So, it is suggested to go with clustering or grouping of the germplasm based on both rhizome yield traits and curcuminoids content together as this kind of clustering results in selection of accessions with high yield along with high curcuminoid content. This kind of clustering will be helpful to find out the superior accessions in a condition where the price is based on curcuminoid content and also on rhizome weight basis. In PCA, PC1, and PC2 together constitute around 45% of the variation. Yield is the major contributor to PC 1 and is confirmed by CL 180 (High yielding accession), which was placed at the extreme right corner of the biplot, and CL 5 (Low yielding accession), which was placed at the extreme left corner of the biplot. Thus, the contrasting accessions were placed in the opposite position to each other. The outcome of PCA was consistent with the result of cluster analysis. Similar results were obtained by Dev et al. (2017) in *Grewia tenax* by using dendrogram and PCA. Cluster analysis and PCA disclosed the complex relationship between the accessions with equal effectiveness (Rezai and Fray, 1990; Shukla et al., 2010).

The weight of secondary rhizomes per plant, weight of mother rhizomes per plant and weight of primary rhizomes per plant contributed maximum to the total divergence. The traits showing the maximum contribution towards divergence can be used further in breeding programs to improve the traits in the existing germplasm. Research findings of Vithya et al. (2021) stated that highest per cent contribution to the total divergence was obtained by weight of fresh rhizome yield per plant (41.99%) followed by weight of primary rhizomes per plant (28.14%) and weight of secondary rhizome per plant (5.63%). However, their conclusions were based on limited number of turmeric genotypes *i.e*., less than 25 and only seven parameters were considered for divergence studies. As the number of traits under study increases, automatically the per cent contribution of each trait to the total divergence decreases. In the present study, 15 rhizome yield traits and four qualitative traits were considered for evaluating the per cent contribution to the total divergence. Thus, the per cent contribution of traits to the total divergence was comparatively low. The selection of parents mainly depends on the contribution of traits towards divergence. The traits showing the maximum contribution towards divergence can be used further in breeding programs to improve the traits in the existing germplasm. Correlation studies were done to identify the relationship between individual traits, especially between total curcuminoid content and rhizome yield traits. Results revealed that total curcuminoid content had a significant positive correlation with primary rhizome core diameter and length of the secondary rhizome. The curcuminoid content is majorly present in the primary rhizome core diameter. So, automatically when the primary rhizome core diameter is more, the percentage of curcuminoid content will be more. A research report on turmeric by Aarthi et al. (2018), also mentioned that curcumin content is highly significant and positively correlated with primary rhizome core diameter. Therefore, selecting these particular rhizome traits may result in the identification of accessions with high curcuminoids content. Similarly, dry recovery percentage is highly significant and positively correlated with the girth of the mother rhizome, and it is highly significant and negatively correlated with primary rhizome core diameter. It indicates that selecting accessions with large mother rhizome girth and smaller primary rhizome core diameter will help in identifying the accessions with a high dry recovery percentage. The yield of the germplasm is highly significant and positively correlated with the weight of primary rhizomes per plant, number of primary rhizomes per plant, and the weight of mother rhizomes per plant. Results obtained by Singh et al. (2018) and Verma et al. (2014) are in line with our results. The present study revealed the diversity of Indian turmeric germplasm in terms of rhizome yield and curcuminoids content in particular it lead to identification of superior germplasm such as CL 157 and CL 272 for curcumin, CL 253 and CL 157 for demethoxycurcumin, CL 216 and CL 57 for bisdemethoxycurcumin and CL 157 and CL 272 for the total curcuminoid content. In addition our findings lead to following recommendations; Firstly, the selection of germplasm should be done based on both rhizome yield traits and curcuminoids content as evident from cluster analysis. Secondly, the advanced analytical techniques like HPTLC and UHPLC can be utilized for precise quantification of total and individual curcuminoids content instead of using traditional spectrophotometric method which will also help in breeding programs and industrial applications.

## Data availability statement

The original contributions presented in the study are included in the article/**Supplementary Material**. Further inquiries can be directed to the corresponding authors.

## Author contributions

MD, SN, VK, RM, and SB contributed to the study conception and design. Material preparation and data collection were done by MD, SN, VK, and SB. Data was analyzed by MD, SS and KA. The first draft of the manuscript was written by MD, KA, BM and SN. All the authors commented on previous versions of the manuscript. All authors contributed to the article and approved the submitted version.
