# Supplementary material for: Unlocking the genetic diversity of Indian turmeric (Curcuma longa L.) germplasm based on rhizome yield traits and curcuminoids
Source: Front Plant Sci. 2022 Dec 15;13:1036592. doi: 10.3389/fpls.2022.1036592 (PMC9797976; doi:10.3389/fpls.2022.1036592)
Supplement: Supplementary file 1 [file DataSheet_1.doc]

| **Supplementary Table 1 |** Origin details of 200 turmeric germplasm accessions. | | |
| --- | --- | --- |
| **Germplasm Code No.** | **Germplasm accessions** | **Origin** |
| CL 1 | BSR – 1 | Tamil Nadu |
| CL 3 | BSR – 3 | Tamil Nadu |
| CL 4 | BSR – 5 | Tamil Nadu |
| CL 5 | BSR – 6 | Tamil Nadu |
| CL 6 | BSR – 7 | Tamil Nadu |
| CL 7 | BSR – 8 | Tamil Nadu |
| CL 8 | BSR – 9 | Tamil Nadu |
| CL 9 | BSR – 10 | Tamil Nadu |
| CL 10 | BSR – 11 | Tamil Nadu |
| CL 12 | BSR – 13 | Tamil Nadu |
| CL 13 | BSR – 14 | Tamil Nadu |
| CL 14 | BSR – 15 | Tamil Nadu |
| CL 16 | BSR – 17 | Tamil Nadu |
| CL 17 | BSR – 18 | Tamil Nadu |
| CL 18 | BSR – 19 | Tamil Nadu |
| CL 19 | BSR – 20 | Tamil Nadu |
| CL 20 | BSR – 21 | Tamil Nadu |
| CL 21 | BSR – 22 | Tamil Nadu |
| CL 23 | BSR – 24 | Tamil Nadu |
| CL 24 | BSR – 25 | Tamil Nadu |
| CL 25 | BSR – 26 | Tamil Nadu |
| CL 26 | BSR – 27 | Tamil Nadu |
| CL 27 | BSR – 28 | Tamil Nadu |
| CL 28 | BSR – 29 | Andhra Pradesh |
| CL 29 | BSR – 30 | Tamil Nadu |
| CL 30 | BSR – 31 | Tamil Nadu |
| CL 32 | BSR – 33 | Andhra Pradesh |
| CL 33 | BSR – 34 | Tamil Nadu |
| CL 34 | BSR – 35 | Tamil Nadu |
| CL 36 | BSR – 37 | Tamil Nadu |
| CL 37 | BSR – 38 | Tamil Nadu |
| CL 38 | BSR – 39 | Tamil Nadu |
| CL 39 | BSR – 40 | Tamil Nadu |
| CL 40 | BSR – 41 | Tamil Nadu |
| CL 44 | BSR – 45 | Tamil Nadu |
| CL 45 | BSR – 46 | Tamil Nadu |
| CL 46 | BSR – 47 | Andhra Pradesh |
| CL 47 | BSR – 48 | Tamil Nadu |
| CL 48 | BSR – 49 | Tamil Nadu |
| CL 50 | BSR – 51 | Tamil Nadu |
| CL 51 | BSR – 52 | Andhra Pradesh |
| CL 53 | BSR – 54 | Tamil Nadu |
| CL 54 | BSR – 55 | Tamil Nadu |
| CL 55 | BSR – 56 | Tamil Nadu |
| CL 56 | BSR – 57 | Tamil Nadu |
| CL 57 | BSR – 58 | Telangana |
| CL 58 | BSR – 59 | Tamil Nadu |
| CL 59 | BSR- 60 | Tamil Nadu |
| CL 60 | BSR – 61 | Tamil Nadu |
| CL 61 | BSR – 62 | Tamil Nadu |
| CL 62 | BSR – 63 | Tamil Nadu |
| CL 63 | BSR – 64 | Gujarat |
| CL 66 | BSR -67 | Andhra Pradesh |
| CL 67 | BSR -68 | Meghalaya |
| CL 68 | BSR -69 | Tamil Nadu |
| CL 69 | BSR -70 | Odisha |
| CL 70 | BSR -71 | Tamil Nadu |
| CL 71 | BSR -72 | Tamil Nadu |
| CL 73 | BSR -74 | Tamil Nadu |
| CL 76 | BSR -77 | Tamil Nadu |
| CL 77 | BSR -78 | Tamil Nadu |
| CL 79 | BSR -80 | Tamil Nadu |
| CL 80 | BSR -81 | Tamil Nadu |
| CL 82 | BSR -83 | Andhra Pradesh |
| CL 83 | BSR -84 | Andhra Pradesh |
| CL 84 | BSR -85 | Kerala |
| CL 86 | BSR -87 | Tamil Nadu |
| CL 87 | BSR -88 | Kerala |
| CL 90 | BSR -91 | Tamil Nadu |
| CL 91 | BSR -92 | Odisha |
| CL 92 | BSR -93 | Madhya Pradesh |
| CL 93 | BSR -94 | Telangana |
| CL 94 | BSR -95 | Tamil Nadu |
| CL 95 | BSR -96 | Tamil Nadu |
| CL 96 | BSR -97 | Tamil Nadu |
| CL 97 | BSR -98 | Tamil Nadu |
| CL 98 | BSR -99 | Tamil Nadu |
| CL 99 | BSR -100 | Tamil Nadu |
| CL 101 | BSR -102 | Odisha |
| CL 102 | BSR -103 | Odisha |
| CL 103 | BSR -104 | Tamil Nadu |
| CL 104 | BSR -105 | Tamil Nadu |
| CL 105 | BSR -106 | Tamil Nadu |
| CL 106 | BSR -107 | Tamil Nadu |
| CL 107 | BSR -108 | Tamil Nadu |
| CL 109 | BSR -110 | Tamil Nadu |
| CL 110 | BSR -111 | Tamil Nadu |
| CL 111 | BSR -112 | Tamil Nadu |
| CL 112 | BSR -113 | Tamil Nadu |
| CL 113 | BSR -114 | Tamil Nadu |
| CL 115 | BSR -116 | Tamil Nadu |
| CL 116 | BSR -117 | Tamil Nadu |
| CL 117 | BSR -118 | Tamil Nadu |
| CL 118 | BSR -119 | Tamil Nadu |
| CL 119 | BSR-120 | Tamil Nadu |
| CL 123 | VK-112 | Kerala |
| CL 124 | VK-116 | Kerala |
| CL 125 | VK-111 | Kerala |
| CL 126 | VK-73 | Kerala |
| CL 127 | VK-76 | Kerala |
| CL 128 | VK-96 | Kerala |
| CL 129 | VK-146 | Kerala |
| CL 136 | Koraput (Local) | Odisha |
| CL 137 | Sunduk (local) | Odisha |
| CL 138 | Prathibha | Odisha |
| CL 139 | PTS-10 | Odisha |
| CL 140 | JTS-2 | Odisha |
| CL 141 | Rajendra Sonia | Odisha |
| CL 143 | Roma | Odisha |
| CL 145 | PTS-62 | Odisha |
| CL 153 | JTS – 1 | Odisha |
| CL 154 | PTS –2 | Tamil Nadu |
| CL 155 | PTS –3 | Tamil Nadu |
| CL 157 | Erode local 1 | Tamil Nadu |
| CL 159 | Erode local 2 | Tamil Nadu |
| CL 161 | PTS-11 | Tamil Nadu |
| CL 162 | Erode local 3 | Tamil Nadu |
| CL 163 | Erode local 4 | Tamil Nadu |
| CL 164 | PTS – 4 | Tamil Nadu |
| CL 165 | Erode local 5 | Tamil Nadu |
| CL 166 | Erode local 6 | Tamil Nadu |
| CL 167 | Erode local 7 | Tamil Nadu |
| CL 168 | PTS 5 | Tamil Nadu |
| CL 171 | Erode local 8 | Tamil Nadu |
| CL 176 | Erode local 9 | Tamil Nadu |
| CL 177 | Erode local 10 | Tamil Nadu |
| CL 178 | Erode local 11 | Tamil Nadu |
| CL 179 | Erode local 12 | Tamil Nadu |
| CL 180 | Erode local 13 | Tamil Nadu |
| CL 181 | Erode local 14 | Tamil Nadu |
| CL 182 | Erode local 15 | Tamil Nadu |
| CL 183 | Erode selection | Tamil Nadu |
| CL 186 | Erode local 16 | Tamil Nadu |
| CL 188 | BDJR – 1250 | Himachal Pradesh |
| CL 191 | RH- 5 | Bihar |
| CL 193 | ACC 360 | Odisha |
| CL 196 | JTS 3 | Telangana |
| CL 197 | PTS-12 | Odisha |
| CL 202 | Anaikatti | Tamil Nadu |
| CL 203 | Erode local 17 | Tamil Nadu |
| CL 204 | Erode local 18 | Tamil Nadu |
| CL 205 | Erode local 19 | Tamil Nadu |
| CL 206 | Erode local 20 | Tamil Nadu |
| CL 207 | Erode local 21 | Tamil Nadu |
| CL 208 | Erode local 22 | Tamil Nadu |
| CL 210 | Erode local 23 | Tamil Nadu |
| CL 211 | Erode local 24 | Tamil Nadu |
| CL 214 | Erode local 25 | Tamil Nadu |
| CL 215 | Erode local 26 | Tamil Nadu |
| CL 216 | Erode local 27 | Tamil Nadu |
| CL 217 | Erode local 28 | Tamil Nadu |
| CL 218 | Erode local 29 | Tamil Nadu |
| CL 219 | Erode local 30 | Tamil Nadu |
| CL 220 | Erode local 31 | Tamil Nadu |
| CL 221 | Erode local 32 | Tamil Nadu |
| CL 222 | Erode local 33 | Tamil Nadu |
| CL 223 | Erode local 34 | Tamil Nadu |
| CL 224 | Rallis Research Center | Karnataka |
| CL 225 | Pallakad local | Kerala |
| CL 226 | Talavadi local 1 | Tamil Nadu |
| CL 227 | Talavadi local 2 | Tamil Nadu |
| CL 229 | Talavadi local 3 | Kerala |
| CL 231 | IC No. 13687 (TCR No. 30) 1 | Kerala |
| CL 232 | IC No. 13674 (TCR No. 36) 1 | Kerala |
| CL 233 | IC No. 70053 (TCR No. 26) 1 | Kerala |
| CL 234 | IC No. 88690 (TCR No. 615) 1 | Kerala |
| CL 235 | IC No. 88738 (TRC No. 738) 1 | Kerala |
| CL 236 | IC No. 136919 (TCR No. 914) 1 | Kerala |
| CL 237 | IC No. 137002 (TCR No. 1245) 1 | Kerala |
| CL 238 | IC No. 70031 (TCR No. 508) 15 | Kerala |
| CL 239 | IC No. 88877 (TCR No. 686) 15 | Kerala |
| CL 240 | IC No. 88788 (TCR No. 754) 15 | Kerala |
| CL 241 | IC No. 38787 (TCR No. 761) 15 | Kerala |
| CL 242 | IC No. 88800 (TCR No. 816) 15 | Kerala |
| CL 243 | IC No. 88911 (TCR No. 889)15 | Kerala |
| CL 244 | IC No. 88923 (TCR No. 906)15 | Kerala |
| CL 245 | IC No. 137105 (TCR No. 926)15 | Kerala |
| CL 246 | IC No. 17177 (TCR No. 436)17 | Kerala |
| CL 247 | IC No. 137115 (TCR No. 1195)21 | Kerala |
| CL 248 | IC No. 137019 (TCR No. 63)15 | Kerala |
| CL 249 | IC No. 266507 (TCR No. 1342)15 | Kerala |
| CL 250 | IC No. 313126 (TCR No. 1345)15 | Kerala |
| CL 251 | IC No. 266518 (TCR No. 1398)UG | Kerala |
| CL 252 | IC No. 210261 (TCR No. 1403)UG | Kerala |
| CL 253 | IC No. 210281 (TCR No. 1422)UG | Kerala |
| CL 254 | IC No. 313542 (TCR No. 1629)UG | Kerala |
| CL 256 | Eraiyur | Tamil Nadu |
| CL 257 | Naiyanur | Tamil Nadu |
| CL 258 | Sorna | Tamil Nadu |
| CL 261 | Attur local 1 | Tamil Nadu |
| CL 265 | Attur local 2 | Tamil Nadu |
| CL 266 | BS-50 | Tamil Nadu |
| CL 267 | BS-100 | Tamil Nadu |
| CL 268 | Suguna | Kerala |
| CL 270 | Prabha | Kerala |
| CL 271 | IISR Prathibha | Kerala |
| CL 272 | Sathyamangalam local | Tamil Nadu |
| CL 273 | Annur local 1 | Tamil Nadu |
| CL 274 | Annur local 2 | Tamil Nadu |
| CL 275 | Sathyamangalam local | Tamil Nadu |

| **Supplementary Table 2 |** Performance of turmeric germplasm based on rhizome yield traits. | | | | | | | | | | | | | | | |
| --- | --- | --- | --- | --- | --- | --- | --- | --- | --- | --- | --- | --- | --- | --- | --- |
| **Germplasm Code No.** | **Yield**  **(g p^-1^)** | **DR**  **(%)** | **MRN** | **PRN** | **SRN** | **MRW**  **(g p^-1^)** | **PRW**  **(g p^-1^)** | **SRW**  **(g p^-1^)** | **MRL**  **(cm)** | **MRG**  **(cm)** | **PRL**  **(cm)** | **PRG**  **(cm)** | **PRD**  **(cm)** | **PRCD**  **(cm)** | **SRL**  **(cm)** |
| **CL-1** | 217.02 | 15.91 | 2.4 | 8.9 | 9.5 | 47.00 | 105.14 | 64.88 | 4.5 | 6.6 | 6.7 | 6.2 | 1.9 | 0.8 | 3.8 |
| **CL-3** | 269.28 | 23.74 | 3.1 | 8.5 | 14.5 | 85.25 | 133.18 | 50.85 | 5.4 | 8.8 | 8.2 | 7.6 | 2.4 | 1.2 | 3.8 |
| **CL-4** | 78.06 | 15.79 | 2.6 | 4.5 | 6.0 | 28.24 | 33.21 | 16.61 | 3.6 | 8.5 | 5.2 | 5.9 | 1.8 | 0.6 | 3.4 |
| **CL-5** | 24.77 | 27.10 | 2.5 | 2.6 | 3.3 | 8.17 | 12.25 | 4.35 | 3.3 | 6.4 | 6.2 | 5.6 | 1.2 | 0.4 | 4.2 |
| **CL-6** | 138.07 | 16.09 | 2.1 | 7.4 | 6.5 | 11.07 | 93.10 | 33.89 | 4.0 | 7.6 | 7.0 | 5.9 | 2.3 | 0.9 | 4.2 |
| **CL-7** | 225.76 | 24.33 | 2.4 | 12.5 | 7.5 | 31.28 | 133.16 | 61.32 | 3.6 | 8.3 | 7.3 | 6.2 | 1.7 | 0.6 | 3.2 |
| **CL-8** | 222.40 | 19.12 | 5.9 | 6.3 | 8.4 | 150.44 | 51.19 | 20.77 | 5.2 | 8.3 | 7.8 | 6.3 | 2.3 | 1.2 | 3.9 |
| **CL-9** | 119.23 | 29.18 | 2.5 | 3.2 | 9.7 | 27.52 | 52.88 | 38.83 | 4.4 | 9.2 | 7.8 | 6.8 | 1.8 | 0.8 | 3.3 |
| **CL-10** | 212.29 | 23.78 | 3.0 | 4.4 | 8.0 | 87.45 | 74.29 | 50.55 | 6.4 | 11.0 | 6.5 | 6.2 | 2.3 | 1.3 | 4.3 |
| **CL-12** | 151.99 | 18.34 | 2.9 | 5.1 | 15.3 | 53.05 | 52.03 | 46.92 | 4.3 | 7.3 | 6.9 | 6.6 | 1.7 | 0.8 | 3.7 |
| **CL-13** | 50.20 | 17.90 | 1.5 | 1.9 | 5.3 | 27.50 | 12.50 | 10.20 | 5.5 | 8.5 | 5.8 | 5.7 | 1.5 | 0.8 | 2.5 |
| **CL-14** | 201.40 | 16.07 | 2.4 | 8.1 | 15.0 | 43.90 | 84.83 | 72.68 | 3.8 | 7.6 | 8.3 | 5.6 | 2.0 | 1.0 | 4.2 |
| **CL-16** | 198.45 | 18.85 | 2.6 | 8.0 | 10.6 | 36.74 | 96.12 | 65.59 | 3.3 | 7.2 | 7.6 | 5.9 | 2.3 | 1.2 | 3.6 |
| **CL-17** | 203.94 | 17.95 | 2.3 | 9.6 | 9.6 | 59.85 | 100.92 | 43.17 | 4.6 | 8.0 | 7.6 | 6.6 | 2.2 | 1.3 | 3.5 |
| **CL-18** | 202.96 | 16.96 | 1.7 | 13.2 | 7.5 | 33.83 | 148.58 | 20.55 | 4.2 | 7.0 | 8.1 | 5.8 | 1.8 | 0.7 | 3.8 |
| **CL-19** | 311.25 | 16.67 | 3.0 | 13.8 | 9.7 | 75.31 | 162.17 | 73.77 | 4.9 | 7.6 | 7.1 | 5.5 | 2.2 | 1.2 | 3.8 |
| **CL-20** | 511.81 | 19.96 | 3.3 | 15.8 | 17.6 | 122.60 | 241.25 | 147.96 | 5.5 | 9.3 | 7.3 | 6.5 | 2.0 | 1.1 | 3.8 |
| **CL-21** | 432.51 | 18.42 | 3.0 | 17.1 | 12.5 | 102.55 | 235.00 | 94.96 | 5.0 | 8.2 | 9.7 | 6.5 | 2.5 | 1.6 | 4.6 |
| **CL-23** | 364.95 | 24.22 | 3.3 | 8.1 | 12.1 | 120.83 | 185.72 | 58.40 | 5.4 | 9.0 | 8.6 | 7.5 | 2.3 | 1.5 | 4.8 |
| **CL-24** | 363.60 | 25.35 | 2.7 | 8.5 | 19.6 | 69.84 | 169.30 | 124.47 | 6.5 | 8.0 | 9.1 | 6.8 | 2.4 | 1.3 | 3.7 |
| **CL-25** | 344.68 | 16.30 | 3.1 | 9.8 | 15.7 | 62.64 | 160.68 | 121.36 | 5.9 | 9.0 | 9.3 | 6.9 | 2.1 | 1.1 | 5.3 |
| **CL-26** | 290.58 | 18.62 | 2.8 | 13.5 | 15.2 | 74.40 | 138.49 | 77.69 | 5.2 | 7.3 | 6.9 | 6.3 | 2.0 | 1.0 | 4.2 |
| **CL-27** | 374.23 | 16.47 | 3.3 | 11.7 | 20.5 | 83.37 | 175.81 | 115.05 | 5.3 | 6.9 | 8.9 | 7.2 | 2.6 | 1.4 | 3.5 |
| **CL-28** | 510.51 | 15.87 | 6.3 | 10.7 | 16.0 | 168.22 | 229.63 | 112.66 | 5.2 | 9.2 | 9.2 | 7.2 | 2.0 | 1.0 | 3.9 |
| **CL-29** | 433.38 | 14.94 | 3.0 | 10.9 | 24.7 | 91.49 | 167.62 | 174.27 | 7.9 | 7.9 | 9.0 | 6.9 | 2.3 | 1.2 | 4.8 |
| **CL-30** | 576.10 | 15.43 | 6.1 | 22.2 | 12.5 | 151.52 | 317.41 | 107.17 | 4.6 | 6.9 | 8.3 | 6.3 | 2.1 | 1.1 | 3.2 |
| **CL-32** | 198.88 | 19.75 | 3.4 | 6.3 | 9.4 | 70.69 | 91.11 | 37.08 | 4.8 | 8.6 | 7.8 | 5.9 | 1.6 | 0.8 | 4.3 |
| **CL-33** | 449.66 | 23.50 | 3.2 | 19.9 | 19.6 | 104.70 | 209.56 | 135.39 | 5.5 | 8.6 | 7.5 | 6.6 | 1.7 | 0.7 | 4.0 |
| **CL-34** | 268.12 | 16.29 | 2.6 | 10.6 | 18.9 | 39.03 | 126.56 | 102.53 | 5.2 | 7.3 | 8.3 | 5.9 | 1.6 | 0.8 | 3.9 |
| **CL-36** | 118.38 | 18.44 | 2.5 | 5.0 | 10.1 | 22.19 | 65.56 | 30.64 | 5.6 | 8.9 | 8.3 | 5.9 | 1.9 | 1.1 | 4.0 |
| **CL-37** | 180.28 | 15.65 | 2.9 | 7.6 | 15.3 | 50.48 | 88.72 | 41.08 | 6.0 | 6.7 | 7.2 | 5.6 | 2.1 | 1.0 | 3.2 |
| **CL-38** | 201.38 | 21.34 | 2.8 | 7.5 | 9.5 | 45.23 | 75.75 | 80.40 | 3.9 | 7.3 | 7.3 | 4.7 | 1.1 | 0.4 | 4.3 |
| **CL-39** | 290.50 | 22.39 | 2.9 | 18.9 | 13.1 | 59.28 | 177.33 | 53.89 | 3.5 | 7.6 | 6.8 | 5.3 | 1.2 | 0.5 | 4.2 |
| **CL-40** | 136.79 | 18.24 | 2.6 | 10.0 | 11.6 | 30.11 | 64.53 | 42.15 | 3.9 | 6.2 | 6.3 | 5.2 | 1.5 | 0.8 | 3.1 |
| **CL-44** | 305.73 | 15.19 | 3.1 | 11.5 | 15.4 | 84.08 | 135.92 | 85.73 | 4.5 | 7.3 | 7.3 | 5.8 | 2.2 | 1.3 | 3.5 |
| **CL-45** | 258.80 | 22.24 | 4.0 | 6.3 | 9.4 | 79.90 | 111.23 | 67.67 | 5.4 | 7.2 | 6.9 | 6.6 | 2.0 | 1.0 | 2.9 |
| **CL-46** | 293.05 | 16.41 | 2.6 | 7.7 | 15.4 | 59.11 | 143.98 | 89.96 | 4.6 | 7.3 | 7.9 | 7.0 | 2.0 | 1.2 | 4.9 |
| **CL-47** | 422.83 | 15.20 | 2.7 | 12.5 | 18.5 | 90.76 | 200.63 | 131.44 | 4.6 | 7.9 | 7.6 | 6.6 | 2.1 | 1.0 | 3.9 |
| **CL-48** | 426.79 | 15.38 | 4.8 | 10.4 | 16.7 | 147.54 | 162.88 | 116.37 | 4.9 | 7.7 | 8.3 | 6.9 | 2.6 | 1.5 | 2.9 |
| **CL-50** | 256.12 | 17.04 | 2.6 | 12.3 | 15.4 | 47.61 | 138.23 | 70.27 | 5.5 | 8.2 | 7.3 | 7.3 | 2.2 | 1.4 | 3.9 |
| **CL-51** | 322.92 | 21.40 | 2.5 | 14.0 | 20.5 | 52.66 | 157.91 | 112.35 | 4.8 | 9.4 | 7.4 | 6.4 | 1.9 | 1.2 | 5.7 |
| **CL-53** | 307.10 | 16.60 | 3.2 | 11.1 | 7.4 | 55.73 | 187.62 | 63.76 | 4.1 | 8.2 | 7.3 | 6.6 | 1.9 | 1.1 | 4.4 |
| **CL-54** | 452.20 | 20.52 | 1.9 | 16.4 | 10.3 | 41.09 | 304.27 | 106.84 | 4.5 | 8.9 | 8.5 | 6.5 | 1.8 | 1.1 | 4.6 |
| **CL-55** | 452.42 | 15.42 | 3.5 | 15.9 | 23.8 | 55.63 | 235.19 | 161.60 | 4.7 | 9.5 | 6.8 | 6.6 | 2.0 | 1.2 | 4.8 |
| **CL-56** | 300.67 | 16.77 | 2.0 | 10.2 | 13.2 | 58.45 | 170.63 | 71.60 | 4.8 | 6.5 | 7.6 | 6.4 | 2.1 | 1.3 | 4.6 |
| **CL-57** | 266.29 | 16.28 | 2.6 | 11.1 | 12.7 | 66.63 | 134.63 | 65.04 | 4.7 | 7.6 | 7.8 | 6.6 | 2.1 | 1.3 | 3.8 |
| **CL-58** | 290.21 | 20.61 | 3.5 | 11.9 | 9.5 | 99.84 | 137.87 | 52.51 | 4.1 | 8.2 | 7.0 | 5.5 | 1.6 | 0.9 | 4.5 |
| **CL-59** | 261.50 | 17.56 | 2.3 | 6.0 | 18.1 | 48.33 | 111.67 | 101.50 | 4.7 | 8.7 | 7.8 | 7.0 | 2.1 | 1.2 | 4.4 |
| **CL-60** | 282.48 | 21.91 | 3.8 | 7.7 | 7.7 | 128.36 | 117.01 | 37.11 | 4.9 | 9.3 | 8.7 | 8.0 | 2.1 | 1.4 | 5.2 |
| **CL-61** | 549.53 | 17.77 | 3.0 | 13.5 | 19.3 | 119.85 | 305.96 | 123.72 | 4.8 | 9.6 | 7.9 | 7.2 | 2.2 | 1.3 | 4.8 |
| **CL-62** | 524.85 | 19.88 | 2.5 | 23.6 | 26.9 | 56.11 | 248.33 | 220.42 | 5.1 | 8.8 | 7.5 | 6.5 | 1.7 | 1.0 | 4.9 |
| **CL-63** | 393.04 | 23.83 | 3.0 | 11.2 | 18.6 | 69.24 | 200.29 | 123.51 | 5.1 | 10.0 | 7.8 | 6.7 | 1.8 | 1.0 | 5.4 |
| **CL-66** | 543.00 | 24.24 | 4.2 | 18.3 | 14.6 | 118.84 | 316.29 | 107.87 | 4.8 | 9.3 | 7.5 | 6.6 | 1.9 | 1.0 | 4.4 |
| **CL-67** | 457.85 | 23.34 | 4.0 | 19.6 | 15.2 | 104.33 | 247.04 | 106.48 | 4.7 | 9.5 | 7.0 | 6.9 | 2.1 | 1.3 | 3.9 |
| **CL-68** | 382.95 | 21.09 | 3.2 | 11.3 | 22.6 | 77.34 | 170.25 | 135.35 | 4.7 | 8.1 | 8.3 | 6.8 | 2.0 | 1.3 | 5.4 |
| **CL-69** | 511.08 | 19.05 | 6.0 | 9.7 | 23.5 | 196.17 | 169.61 | 145.31 | 4.9 | 10.2 | 6.5 | 6.5 | 1.8 | 1.0 | 3.5 |
| **CL-70** | 499.08 | 20.91 | 7.0 | 19.3 | 16.1 | 160.77 | 235.29 | 103.02 | 4.5 | 10.0 | 6.7 | 5.8 | 1.7 | 0.9 | 4.1 |
| **CL-71** | 407.59 | 18.05 | 4.6 | 16.0 | 16.0 | 98.55 | 247.78 | 61.26 | 4.9 | 7.9 | 8.3 | 5.8 | 2.3 | 1.2 | 3.9 |
| **CL-73** | 420.87 | 19.96 | 3.7 | 12.5 | 13.6 | 115.39 | 224.33 | 81.15 | 5.6 | 7.6 | 7.9 | 6.3 | 2.2 | 1.2 | 3.6 |
| **CL-76** | 433.31 | 15.92 | 3.9 | 13.6 | 9.1 | 109.68 | 234.95 | 88.67 | 5.1 | 9.8 | 7.2 | 6.8 | 1.9 | 1.3 | 5.2 |
| **CL-77** | 300.03 | 23.21 | 3.5 | 8.1 | 15.0 | 79.79 | 150.85 | 69.38 | 5.8 | 7.6 | 8.9 | 7.7 | 2.3 | 1.5 | 3.6 |
| **CL-79** | 413.00 | 15.82 | 3.1 | 14.6 | 16.4 | 108.38 | 172.82 | 131.81 | 4.7 | 7.7 | 7.7 | 6.1 | 2.0 | 0.9 | 4.0 |
| **CL-80** | 429.65 | 19.96 | 3.2 | 14.0 | 16.1 | 87.30 | 231.96 | 110.39 | 5.9 | 7.6 | 8.7 | 6.2 | 1.7 | 0.7 | 4.1 |
| **CL-82** | 419.80 | 18.90 | 2.6 | 9.5 | 26.6 | 78.62 | 217.29 | 123.89 | 5.7 | 8.8 | 9.2 | 7.0 | 2.6 | 1.7 | 4.4 |
| **CL-83** | 320.56 | 22.16 | 2.2 | 13.7 | 6.3 | 50.43 | 210.61 | 59.52 | 4.8 | 7.0 | 8.3 | 7.0 | 2.3 | 1.3 | 3.0 |
| **CL-84** | 350.04 | 15.18 | 3.3 | 14.0 | 11.2 | 88.54 | 193.93 | 67.57 | 4.5 | 7.3 | 8.0 | 6.2 | 2.6 | 1.6 | 3.5 |
| **CL-86** | 264.16 | 17.67 | 2.5 | 5.8 | 11.6 | 69.34 | 128.95 | 65.88 | 6.2 | 9.3 | 8.3 | 7.0 | 2.6 | 1.7 | 4.3 |
| **CL-87** | 265.97 | 17.35 | 2.9 | 8.9 | 12.5 | 57.02 | 155.49 | 53.46 | 4.9 | 6.9 | 8.0 | 6.9 | 2.4 | 1.3 | 3.9 |
| **CL-90** | 359.39 | 22.98 | 2.6 | 12.1 | 25.8 | 51.09 | 231.66 | 76.64 | 5.9 | 11.0 | 8.4 | 6.2 | 2.8 | 1.6 | 3.8 |
| **CL-91** | 460.44 | 21.93 | 4.1 | 18.6 | 21.4 | 89.51 | 253.93 | 116.99 | 5.8 | 8.2 | 10.3 | 6.6 | 2.5 | 1.5 | 4.0 |
| **CL-92** | 299.73 | 15.60 | 2.0 | 8.4 | 12.6 | 63.90 | 161.71 | 74.12 | 5.6 | 8.3 | 8.6 | 6.6 | 2.5 | 1.5 | 4.1 |
| **CL-93** | 257.82 | 20.95 | 2.0 | 9.3 | 8.6 | 41.92 | 153.03 | 62.88 | 4.2 | 7.1 | 8.9 | 6.6 | 2.3 | 1.2 | 2.9 |
| **CL-94** | 360.81 | 15.22 | 3.0 | 9.1 | 12.6 | 93.93 | 194.46 | 72.43 | 5.9 | 7.3 | 7.6 | 6.0 | 2.5 | 1.6 | 2.9 |
| **CL-95** | 183.61 | 18.53 | 3.2 | 9.2 | 16.1 | 61.93 | 89.56 | 32.11 | 3.7 | 6.8 | 5.5 | 6.4 | 1.9 | 0.9 | 3.7 |
| **CL-96** | 427.06 | 18.70 | 2.9 | 18.0 | 9.1 | 76.18 | 268.63 | 82.25 | 5.0 | 8.6 | 7.7 | 5.8 | 1.6 | 0.7 | 3.5 |
| **CL-97** | 527.26 | 22.30 | 4.2 | 22.6 | 22.3 | 86.80 | 276.57 | 163.89 | 6.2 | 9.0 | 8.4 | 6.7 | 2.5 | 1.2 | 3.9 |
| **CL-98** | 472.87 | 16.73 | 4.9 | 11.2 | 11.2 | 142.94 | 238.35 | 91.57 | 4.9 | 7.7 | 8.9 | 6.0 | 2.3 | 1.2 | 4.5 |
| **CL-99** | 548.59 | 22.27 | 3.1 | 16.7 | 30.6 | 68.89 | 252.17 | 227.53 | 5.3 | 9.6 | 9.0 | 7.3 | 2.3 | 1.3 | 3.2 |
| **CL-101** | 438.57 | 21.54 | 6.8 | 13.2 | 19.9 | 163.25 | 169.43 | 105.89 | 5.6 | 10.6 | 8.9 | 6.0 | 2.1 | 1.0 | 3.6 |
| **CL-102** | 325.40 | 15.66 | 4.5 | 10.9 | 13.1 | 107.04 | 168.11 | 50.24 | 4.9 | 7.4 | 7.7 | 6.6 | 2.2 | 1.4 | 3.5 |
| **CL-103** | 248.77 | 16.06 | 3.4 | 9.9 | 15.5 | 90.64 | 114.37 | 43.77 | 5.0 | 6.2 | 6.7 | 6.3 | 2.3 | 1.3 | 3.3 |
| **CL-104** | 370.66 | 22.91 | 3.7 | 12.9 | 21.5 | 90.21 | 174.96 | 105.49 | 4.9 | 8.0 | 7.4 | 5.5 | 1.5 | 0.5 | 3.9 |
| **CL-105** | 288.11 | 17.09 | 2.7 | 5.4 | 10.7 | 91.23 | 121.75 | 75.13 | 4.6 | 8.2 | 7.3 | 6.6 | 2.3 | 1.4 | 4.2 |
| **CL-106** | 387.42 | 21.59 | 3.8 | 16.0 | 12.8 | 115.29 | 217.69 | 54.44 | 5.7 | 8.0 | 7.6 | 5.6 | 2.0 | 1.1 | 3.3 |
| **CL-107** | 515.65 | 23.84 | 5.8 | 18.6 | 18.5 | 186.19 | 191.15 | 138.31 | 6.3 | 9.2 | 9.3 | 6.2 | 2.3 | 1.2 | 3.6 |
| **CL-109** | 368.08 | 15.57 | 3.0 | 10.3 | 14.5 | 113.79 | 169.53 | 84.76 | 4.9 | 8.2 | 8.0 | 6.8 | 2.2 | 1.3 | 2.9 |
| **CL-110** | 297.60 | 20.93 | 3.6 | 13.9 | 10.9 | 67.32 | 172.86 | 57.42 | 5.5 | 7.3 | 7.0 | 6.9 | 1.9 | 1.1 | 3.9 |
| **CL-111** | 265.74 | 24.15 | 4.0 | 11.4 | 14.3 | 125.85 | 91.53 | 48.37 | 5.6 | 8.6 | 7.6 | 5.9 | 2.0 | 0.9 | 3.6 |
| **CL-112** | 251.04 | 24.81 | 3.3 | 7.2 | 9.0 | 113.54 | 61.81 | 75.69 | 5.9 | 9.7 | 6.7 | 5.9 | 1.8 | 1.0 | 3.4 |
| **CL-113** | 322.44 | 25.65 | 2.8 | 11.2 | 21.9 | 78.35 | 126.13 | 117.97 | 5.2 | 7.0 | 8.5 | 5.9 | 2.1 | 1.2 | 4.2 |
| **CL-115** | 516.12 | 15.28 | 4.8 | 16.1 | 19.6 | 147.28 | 276.03 | 92.81 | 5.6 | 8.7 | 8.9 | 6.5 | 2.4 | 1.4 | 3.5 |
| **CL-116** | 300.46 | 17.63 | 7.7 | 6.1 | 6.1 | 201.69 | 77.39 | 21.39 | 6.0 | 9.3 | 6.2 | 5.2 | 2.3 | 1.0 | 3.5 |
| **CL-117** | 343.85 | 21.51 | 2.7 | 20.5 | 14.2 | 86.66 | 159.50 | 97.69 | 6.3 | 8.6 | 7.9 | 6.0 | 1.6 | 0.8 | 3.3 |
| **CL-118** | 408.28 | 25.11 | 3.5 | 9.0 | 18.0 | 90.17 | 165.96 | 152.15 | 5.5 | 9.8 | 9.5 | 7.2 | 2.2 | 1.4 | 6.0 |
| **CL-119** | 430.23 | 14.85 | 4.8 | 17.7 | 11.8 | 107.99 | 282.97 | 39.27 | 5.6 | 8.9 | 9.2 | 6.5 | 2.3 | 1.4 | 3.2 |
| **CL-123** | 431.88 | 16.96 | 3.2 | 14.4 | 21.5 | 55.86 | 246.57 | 129.45 | 5.4 | 9.0 | 8.8 | 7.6 | 2.4 | 1.6 | 5.9 |
| **CL-124** | 360.90 | 24.30 | 3.0 | 8.4 | 21.0 | 69.16 | 167.91 | 123.83 | 6.3 | 7.9 | 8.8 | 6.7 | 2.3 | 1.2 | 3.6 |
| **CL-125** | 517.44 | 17.82 | 3.8 | 13.7 | 19.7 | 145.06 | 217.52 | 154.86 | 6.2 | 9.5 | 8.7 | 5.2 | 1.8 | 1.0 | 4.2 |
| **CL-126** | 439.37 | 19.35 | 3.1 | 18.2 | 13.9 | 79.95 | 259.84 | 99.58 | 4.3 | 9.9 | 7.9 | 6.8 | 1.8 | 1.1 | 3.3 |
| **CL-127** | 429.06 | 22.79 | 5.5 | 24.2 | 9.2 | 130.30 | 248.09 | 50.67 | 4.1 | 8.6 | 6.8 | 6.2 | 1.6 | 1.0 | 3.0 |
| **CL-128** | 405.05 | 22.69 | 2.8 | 14.5 | 14.5 | 102.46 | 248.36 | 54.24 | 4.4 | 9.1 | 6.5 | 5.8 | 1.6 | 0.9 | 4.0 |
| **CL-129** | 491.75 | 21.82 | 6.1 | 16.4 | 14.2 | 192.45 | 202.95 | 96.35 | 5.7 | 9.0 | 9.6 | 6.5 | 2.3 | 1.2 | 4.2 |
| **CL-136** | 436.59 | 23.09 | 3.0 | 19.0 | 15.5 | 72.93 | 270.36 | 93.29 | 3.8 | 9.3 | 7.6 | 6.0 | 1.7 | 0.9 | 4.1 |
| **CL-137** | 548.90 | 22.22 | 3.8 | 19.2 | 14.3 | 108.66 | 382.51 | 57.74 | 5.3 | 12.2 | 7.6 | 6.6 | 1.7 | 1.0 | 3.4 |
| **CL-138** | 497.91 | 21.41 | 5.2 | 19.1 | 14.5 | 133.94 | 258.73 | 105.24 | 5.8 | 9.7 | 8.2 | 6.7 | 2.2 | 1.2 | 4.1 |
| **CL-139** | 394.97 | 24.21 | 4.1 | 8.5 | 6.8 | 124.45 | 230.14 | 40.38 | 5.9 | 8.0 | 8.6 | 6.5 | 2.5 | 1.3 | 3.9 |
| **CL-140** | 373.08 | 22.61 | 2.9 | 15.3 | 19.7 | 52.42 | 235.47 | 85.18 | 5.2 | 10.8 | 8.3 | 7.9 | 1.6 | 1.0 | 2.6 |
| **CL-141** | 325.19 | 15.83 | 3.9 | 16.2 | 7.5 | 84.21 | 179.83 | 61.14 | 4.2 | 7.8 | 7.3 | 6.4 | 2.0 | 1.1 | 2.7 |
| **CL-143** | 550.04 | 22.31 | 3.4 | 10.2 | 23.8 | 124.34 | 303.10 | 122.60 | 4.6 | 9.8 | 8.5 | 7.2 | 2.2 | 1.5 | 4.9 |
| **CL-145** | 380.19 | 24.76 | 4.0 | 11.9 | 8.1 | 89.06 | 219.88 | 71.25 | 4.1 | 8.2 | 7.4 | 6.8 | 1.8 | 1.2 | 3.5 |
| **CL-153** | 446.14 | 22.39 | 3.8 | 22.6 | 10.9 | 123.89 | 265.62 | 56.63 | 4.3 | 9.4 | 6.5 | 6.1 | 1.7 | 0.9 | 3.3 |
| **CL-154** | 314.67 | 26.57 | 3.0 | 10.4 | 11.7 | 77.78 | 177.26 | 59.63 | 4.7 | 8.1 | 9.2 | 7.4 | 2.1 | 1.2 | 3.1 |
| **CL-155** | 358.74 | 15.17 | 3.0 | 15.5 | 4.6 | 77.95 | 238.31 | 42.48 | 4.8 | 9.7 | 8.1 | 7.5 | 2.1 | 1.2 | 3.6 |
| **CL-157** | 359.32 | 24.38 | 3.1 | 16.9 | 19.7 | 106.73 | 185.19 | 67.41 | 5.5 | 10.3 | 8.3 | 7.2 | 2.5 | 1.7 | 4.9 |
| **CL-159** | 356.24 | 16.26 | 5.0 | 16.2 | 13.5 | 134.01 | 178.68 | 43.56 | 4.9 | 8.5 | 6.0 | 6.5 | 1.8 | 1.1 | 3.6 |
| **CL-161** | 620.08 | 24.85 | 5.9 | 11.7 | 12.5 | 229.28 | 297.21 | 93.58 | 5.7 | 9.1 | 7.6 | 7.9 | 2.5 | 1.3 | 3.5 |
| **CL-162** | 529.35 | 25.86 | 6.0 | 16.8 | 6.5 | 175.53 | 305.73 | 48.09 | 4.7 | 10.0 | 7.8 | 7.1 | 2.1 | 1.2 | 3.2 |
| **CL-163** | 437.73 | 17.32 | 4.0 | 13.5 | 9.5 | 128.25 | 231.68 | 77.79 | 5.9 | 9.6 | 8.4 | 6.6 | 2.3 | 1.4 | 3.4 |
| **CL-164** | 520.11 | 14.95 | 2.5 | 18.4 | 15.3 | 82.84 | 336.01 | 101.25 | 5.1 | 9.3 | 6.9 | 7.0 | 2.0 | 1.4 | 3.5 |
| **CL-165** | 529.98 | 24.17 | 6.5 | 13.2 | 9.6 | 199.20 | 270.64 | 60.14 | 4.7 | 9.2 | 7.3 | 7.3 | 2.1 | 1.0 | 2.9 |
| **CL-166** | 412.62 | 14.89 | 4.5 | 17.0 | 15.5 | 113.14 | 224.61 | 74.87 | 5.1 | 9.3 | 6.8 | 7.4 | 2.0 | 1.3 | 3.5 |
| **CL-167** | 359.62 | 22.74 | 2.8 | 21.6 | 8.5 | 66.50 | 224.34 | 68.79 | 3.9 | 7.9 | 6.5 | 5.0 | 1.3 | 0.8 | 3.6 |
| **CL-168** | 488.28 | 23.85 | 3.5 | 22.1 | 18.7 | 79.72 | 322.06 | 86.51 | 5.8 | 9.1 | 7.7 | 6.0 | 1.5 | 0.9 | 3.7 |
| **CL-171** | 658.15 | 22.20 | 6.5 | 24.2 | 23.6 | 152.97 | 354.65 | 150.54 | 4.9 | 10.1 | 8.4 | 6.4 | 1.8 | 1.0 | 3.5 |
| **CL-176** | 496.83 | 24.38 | 6.8 | 15.8 | 10.1 | 156.27 | 253.52 | 87.04 | 4.0 | 9.2 | 6.5 | 6.1 | 1.9 | 1.0 | 4.3 |
| **CL-177** | 365.44 | 14.96 | 3.6 | 12.4 | 10.4 | 111.77 | 192.50 | 61.17 | 4.2 | 8.5 | 7.8 | 7.0 | 2.1 | 1.2 | 3.9 |
| **CL-178** | 420.89 | 24.56 | 3.8 | 14.1 | 11.6 | 104.64 | 231.65 | 84.60 | 4.6 | 8.9 | 8.0 | 7.4 | 2.3 | 1.3 | 3.7 |
| **CL-179** | 305.74 | 24.52 | 4.1 | 8.9 | 9.6 | 86.83 | 187.75 | 31.17 | 5.2 | 9.6 | 7.4 | 6.7 | 1.7 | 1.0 | 3.7 |
| **CL-180** | 667.63 | 23.33 | 3.0 | 23.5 | 22.5 | 122.48 | 392.50 | 152.64 | 5.6 | 10.7 | 8.0 | 7.5 | 1.9 | 1.2 | 4.2 |
| **CL-181** | 454.01 | 25.00 | 4.9 | 17.9 | 14.3 | 137.63 | 264.54 | 51.83 | 4.8 | 10.1 | 8.3 | 6.5 | 1.9 | 1.2 | 3.7 |
| **CL-182** | 415.37 | 14.72 | 7.3 | 10.2 | 14.0 | 218.96 | 155.67 | 40.74 | 4.8 | 9.2 | 7.4 | 6.1 | 1.8 | 1.0 | 4.0 |
| **CL-183** | 362.32 | 22.87 | 2.7 | 14.2 | 19.5 | 76.30 | 163.25 | 122.76 | 6.2 | 9.0 | 7.3 | 6.2 | 2.2 | 1.1 | 3.9 |
| **CL-186** | 160.86 | 25.54 | 2.6 | 6.8 | 10.9 | 46.14 | 82.57 | 32.14 | 5.2 | 9.7 | 8.3 | 6.6 | 2.1 | 1.0 | 3.6 |
| **CL-188** | 531.66 | 25.77 | 6.5 | 13.9 | 11.6 | 187.59 | 262.44 | 81.64 | 6.8 | 9.3 | 9.0 | 7.1 | 2.5 | 1.6 | 3.8 |
| **CL-191** | 304.55 | 13.67 | 7.6 | 13.5 | 15.1 | 156.58 | 102.55 | 45.42 | 4.4 | 8.2 | 6.7 | 6.1 | 1.8 | 1.2 | 2.5 |
| **CL-193** | 238.29 | 13.87 | 2.9 | 11.5 | 11.5 | 39.29 | 166.26 | 32.74 | 4.5 | 7.3 | 7.2 | 6.8 | 1.8 | 1.1 | 3.6 |
| **CL-196** | 505.94 | 27.22 | 6.2 | 21.2 | 11.7 | 166.60 | 264.45 | 74.89 | 4.5 | 9.1 | 7.5 | 6.6 | 2.0 | 1.1 | 3.7 |
| **CL-197** | 323.63 | 14.16 | 3.1 | 12.4 | 10.4 | 68.33 | 215.33 | 39.97 | 5.6 | 8.7 | 8.1 | 7.6 | 2.3 | 1.5 | 4.8 |
| **CL-202** | 304.72 | 21.83 | 4.2 | 12.2 | 8.9 | 86.54 | 196.38 | 21.80 | 5.9 | 8.6 | 9.0 | 6.7 | 2.9 | 1.9 | 2.8 |
| **CL-203** | 253.07 | 23.75 | 3.1 | 7.1 | 14.6 | 80.05 | 125.85 | 47.17 | 5.6 | 9.1 | 8.3 | 5.7 | 2.2 | 1.3 | 3.6 |
| **CL-204** | 244.36 | 24.49 | 1.8 | 14.9 | 14.9 | 39.57 | 155.14 | 49.64 | 5.7 | 9.3 | 9.3 | 5.5 | 1.9 | 1.0 | 3.9 |
| **CL-205** | 346.86 | 24.24 | 4.2 | 8.6 | 10.5 | 99.29 | 148.29 | 99.29 | 5.6 | 6.6 | 8.6 | 6.6 | 2.1 | 1.0 | 4.3 |
| **CL-206** | 357.03 | 22.86 | 3.0 | 12.3 | 18.6 | 55.87 | 207.15 | 94.02 | 4.9 | 7.9 | 8.3 | 7.0 | 2.5 | 1.6 | 4.6 |
| **CL-207** | 471.23 | 27.01 | 5.7 | 13.1 | 16.5 | 167.64 | 217.14 | 86.44 | 6.9 | 9.2 | 8.6 | 6.8 | 2.8 | 1.7 | 3.8 |
| **CL-208** | 328.92 | 16.73 | 2.7 | 8.9 | 13.3 | 63.24 | 188.03 | 77.66 | 5.4 | 8.7 | 7.9 | 5.9 | 1.9 | 1.0 | 4.6 |
| **CL-210** | 490.91 | 21.95 | 4.8 | 13.9 | 20.1 | 147.71 | 206.23 | 136.97 | 4.5 | 8.9 | 9.0 | 5.9 | 2.2 | 1.1 | 4.2 |
| **CL-211** | 451.31 | 23.64 | 2.9 | 21.6 | 19.6 | 66.74 | 280.52 | 104.04 | 5.4 | 8.9 | 8.6 | 6.5 | 2.1 | 1.0 | 4.0 |
| **CL-214** | 435.82 | 17.38 | 4.0 | 6.4 | 14.9 | 135.96 | 191.51 | 108.34 | 4.5 | 7.4 | 9.1 | 7.0 | 2.5 | 1.4 | 4.3 |
| **CL-215** | 381.42 | 22.99 | 3.4 | 18.8 | 8.5 | 114.87 | 208.61 | 57.95 | 4.8 | 9.8 | 7.6 | 5.8 | 2.0 | 1.2 | 2.5 |
| **CL-216** | 398.14 | 15.65 | 3.5 | 22.9 | 10.5 | 101.69 | 264.89 | 31.56 | 5.0 | 7.6 | 9.1 | 7.6 | 2.5 | 1.6 | 3.3 |
| **CL-217** | 356.86 | 20.14 | 2.9 | 20.4 | 10.8 | 83.30 | 193.66 | 79.90 | 4.1 | 7.9 | 8.0 | 5.2 | 1.7 | 0.7 | 3.6 |
| **CL-218** | 441.07 | 18.55 | 4.6 | 11.0 | 21.5 | 149.53 | 182.52 | 109.02 | 5.9 | 9.7 | 8.7 | 6.3 | 2.3 | 1.3 | 3.5 |
| **CL-219** | 281.63 | 21.97 | 2.0 | 9.3 | 10.3 | 43.89 | 188.25 | 49.49 | 4.9 | 8.4 | 10.0 | 7.2 | 2.6 | 1.5 | 3.2 |
| **CL-220** | 235.42 | 13.42 | 1.6 | 11.2 | 8.7 | 40.99 | 167.11 | 27.33 | 5.0 | 7.5 | 8.7 | 6.8 | 2.5 | 1.4 | 3.6 |
| **CL-221** | 368.31 | 14.13 | 3.5 | 10.4 | 13.0 | 93.42 | 210.01 | 64.88 | 5.5 | 8.3 | 9.2 | 6.3 | 2.6 | 1.8 | 3.7 |
| **CL-222** | 410.52 | 14.66 | 3.0 | 14.1 | 19.7 | 81.64 | 230.36 | 98.53 | 5.2 | 6.6 | 9.3 | 7.3 | 2.5 | 1.7 | 4.6 |
| **CL-223** | 326.18 | 22.96 | 2.6 | 8.4 | 15.6 | 73.38 | 158.97 | 93.83 | 5.5 | 8.9 | 9.9 | 7.3 | 2.8 | 1.7 | 5.1 |
| **CL-224** | 339.72 | 15.42 | 3.6 | 13.9 | 13.9 | 91.97 | 200.38 | 47.38 | 5.2 | 8.0 | 7.1 | 6.0 | 1.9 | 1.1 | 3.5 |
| **CL-225** | 355.77 | 15.42 | 2.6 | 5.6 | 14.9 | 85.74 | 137.70 | 132.34 | 5.1 | 8.7 | 8.5 | 7.5 | 2.4 | 1.7 | 6.0 |
| **CL-226** | 455.49 | 18.92 | 2.4 | 19.5 | 11.7 | 78.11 | 320.75 | 56.63 | 6.2 | 8.7 | 8.7 | 6.7 | 2.3 | 1.3 | 4.3 |
| **CL-227** | 454.91 | 15.13 | 2.6 | 13.6 | 15.5 | 72.72 | 261.75 | 120.44 | 4.2 | 8.3 | 8.2 | 6.9 | 2.3 | 1.5 | 3.0 |
| **CL-229** | 532.25 | 14.56 | 6.5 | 13.4 | 9.5 | 174.14 | 296.87 | 61.24 | 5.0 | 8.9 | 8.9 | 7.6 | 2.9 | 2.0 | 3.0 |
| **CL-231** | 282.05 | 25.36 | 3.6 | 11.8 | 7.1 | 61.76 | 161.16 | 59.13 | 5.8 | 7.3 | 7.5 | 5.9 | 2.0 | 0.7 | 3.4 |
| **CL-232** | 276.13 | 23.72 | 3.0 | 6.1 | 15.4 | 125.96 | 96.78 | 53.39 | 5.7 | 8.0 | 7.6 | 5.7 | 2.3 | 1.2 | 3.2 |
| **CL-233** | 459.20 | 13.98 | 3.9 | 20.1 | 9.5 | 76.58 | 326.09 | 56.53 | 4.5 | 8.3 | 8.4 | 6.4 | 2.1 | 1.2 | 2.9 |
| **CL-234** | 445.48 | 24.95 | 3.9 | 15.1 | 9.6 | 137.37 | 226.23 | 81.87 | 4.6 | 8.3 | 7.6 | 5.9 | 2.2 | 1.2 | 3.6 |
| **CL-235** | 475.63 | 20.35 | 3.0 | 12.6 | 16.2 | 93.04 | 296.38 | 86.22 | 6.0 | 8.6 | 8.2 | 7.5 | 2.6 | 1.7 | 3.0 |
| **CL-236** | 401.12 | 20.80 | 2.6 | 21.2 | 10.5 | 71.02 | 248.39 | 81.71 | 4.8 | 8.7 | 8.8 | 7.2 | 2.1 | 1.1 | 3.2 |
| **CL-237** | 235.62 | 15.34 | 2.4 | 10.8 | 7.8 | 60.71 | 148.46 | 26.44 | 5.0 | 8.6 | 7.9 | 7.8 | 2.7 | 1.9 | 3.3 |
| **CL-238** | 409.42 | 23.50 | 3.1 | 15.4 | 10.3 | 94.58 | 285.00 | 29.84 | 5.6 | 9.4 | 8.2 | 6.8 | 2.4 | 1.5 | 2.9 |
| **CL-239** | 303.15 | 22.83 | 2.9 | 17.7 | 5.2 | 72.92 | 167.16 | 63.06 | 4.6 | 7.3 | 8.8 | 5.7 | 1.9 | 0.9 | 3.2 |
| **CL-240** | 176.12 | 16.41 | 2.6 | 7.9 | 11.9 | 45.24 | 97.17 | 33.71 | 4.4 | 8.3 | 7.5 | 5.8 | 2.2 | 1.2 | 3.6 |
| **CL-241** | 367.89 | 14.20 | 3.5 | 10.1 | 4.5 | 90.40 | 213.64 | 63.85 | 5.6 | 8.3 | 8.4 | 7.6 | 2.4 | 1.5 | 3.6 |
| **CL-242** | 588.09 | 26.27 | 4.6 | 24.6 | 15.4 | 135.97 | 405.93 | 46.18 | 5.3 | 9.6 | 8.2 | 6.6 | 2.2 | 1.4 | 2.8 |
| **CL-243** | 395.58 | 26.53 | 2.7 | 15.2 | 11.4 | 79.44 | 243.46 | 72.68 | 4.5 | 9.2 | 8.1 | 6.4 | 2.2 | 1.1 | 3.4 |
| **CL-244** | 108.09 | 23.88 | 1.6 | 7.6 | 7.6 | 25.83 | 62.58 | 19.68 | 5.3 | 9.1 | 6.7 | 4.7 | 1.3 | 0.6 | 3.1 |
| **CL-245** | 269.88 | 16.01 | 2.2 | 9.7 | 9.6 | 58.55 | 162.86 | 48.47 | 4.5 | 7.6 | 8.3 | 6.5 | 2.5 | 1.6 | 3.3 |
| **CL-246** | 408.86 | 20.78 | 4.0 | 20.8 | 13.5 | 111.26 | 219.03 | 78.57 | 4.7 | 7.9 | 7.8 | 5.4 | 1.7 | 0.7 | 3.5 |
| **CL-247** | 228.98 | 25.70 | 3.9 | 7.0 | 8.9 | 54.64 | 133.61 | 40.73 | 4.6 | 9.2 | 8.6 | 6.9 | 2.2 | 1.3 | 3.9 |
| **CL-248** | 147.03 | 14.84 | 2.2 | 6.4 | 5.2 | 45.16 | 74.79 | 27.08 | 5.0 | 8.5 | 6.6 | 5.8 | 2.3 | 1.3 | 3.7 |
| **CL-249** | 295.16 | 21.41 | 2.1 | 8.9 | 10.9 | 60.28 | 166.68 | 68.19 | 6.4 | 8.7 | 7.2 | 7.5 | 2.6 | 1.7 | 4.1 |
| **CL-250** | 282.35 | 16.16 | 2.4 | 12.0 | 9.8 | 51.17 | 186.53 | 44.64 | 3.9 | 7.5 | 9.3 | 5.3 | 2.2 | 1.3 | 4.2 |
| **CL-251** | 219.16 | 16.41 | 1.5 | 7.3 | 8.2 | 32.65 | 147.50 | 39.00 | 4.9 | 8.8 | 8.5 | 6.8 | 2.4 | 1.5 | 3.4 |
| **CL-252** | 334.87 | 25.38 | 3.0 | 16.6 | 14.7 | 37.15 | 214.60 | 83.12 | 4.7 | 8.5 | 8.8 | 6.8 | 2.2 | 1.2 | 3.9 |
| **CL-253** | 599.14 | 22.75 | 3.6 | 23.5 | 21.7 | 85.29 | 375.88 | 137.98 | 6.1 | 8.0 | 8.9 | 6.4 | 2.3 | 1.4 | 3.5 |
| **CL-254** | 302.50 | 21.09 | 2.6 | 16.5 | 14.0 | 52.52 | 206.83 | 43.14 | 4.3 | 8.7 | 7.9 | 6.2 | 2.1 | 1.0 | 3.2 |
| **CL-256** | 280.86 | 14.43 | 4.9 | 10.4 | 10.4 | 115.83 | 120.43 | 44.60 | 5.6 | 6.8 | 8.6 | 6.9 | 2.3 | 1.2 | 3.6 |
| **CL-257** | 157.14 | 14.63 | 1.6 | 7.9 | 4.8 | 35.82 | 100.70 | 20.62 | 5.4 | 8.1 | 8.2 | 6.1 | 2.1 | 1.0 | 2.7 |
| **CL-258** | 438.98 | 26.83 | 5.5 | 20.3 | 10.1 | 168.85 | 192.46 | 77.67 | 5.2 | 9.1 | 7.4 | 7.7 | 2.3 | 1.4 | 6.0 |
| **CL-261** | 383.29 | 15.38 | 3.6 | 12.2 | 19.8 | 86.98 | 221.55 | 74.77 | 4.8 | 8.1 | 8.3 | 7.2 | 2.4 | 1.5 | 2.9 |
| **CL-265** | 296.32 | 23.24 | 3.1 | 21.3 | 9.9 | 72.35 | 202.68 | 21.28 | 4.9 | 8.3 | 7.6 | 6.3 | 2.1 | 1.1 | 3.2 |
| **CL-266** | 278.27 | 15.06 | 3.5 | 12.1 | 9.4 | 54.66 | 180.76 | 42.85 | 5.6 | 7.6 | 7.7 | 6.9 | 2.4 | 1.4 | 3.9 |
| **CL-267** | 186.41 | 22.03 | 2.7 | 7.8 | 12.6 | 74.96 | 86.33 | 25.11 | 4.6 | 8.3 | 6.2 | 5.3 | 2.0 | 0.9 | 2.9 |
| **CL-268** | 191.22 | 22.94 | 2.5 | 11.4 | 8.0 | 60.14 | 111.71 | 19.38 | 4.6 | 6.6 | 7.3 | 5.2 | 2.0 | 1.1 | 2.9 |
| **CL-270** | 293.93 | 22.92 | 3.5 | 9.1 | 8.2 | 102.14 | 166.34 | 25.45 | 6.0 | 8.6 | 9.9 | 7.8 | 2.8 | 1.8 | 3.0 |
| **CL-271** | 243.34 | 15.94 | 3.5 | 8.7 | 6.3 | 70.20 | 147.05 | 26.09 | 4.3 | 6.1 | 7.9 | 6.7 | 1.9 | 1.0 | 3.8 |
| **CL-272** | 362.26 | 23.25 | 3.7 | 10.6 | 13.3 | 114.46 | 165.29 | 82.51 | 4.9 | 9.7 | 9.4 | 7.4 | 2.2 | 1.2 | 5.8 |
| **CL-273** | 471.18 | 21.29 | 7.5 | 15.8 | 10.1 | 155.86 | 234.51 | 80.80 | 5.9 | 8.8 | 8.6 | 6.4 | 2.2 | 1.2 | 3.6 |
| **CL-274** | 381.54 | 15.26 | 3.6 | 14.2 | 10.5 | 82.75 | 203.94 | 94.85 | 5.5 | 9.0 | 8.3 | 7.3 | 2.4 | 1.6 | 4.2 |
| **CL-275** | 388.07 | 23.26 | 2.6 | 18.9 | 18.5 | 69.30 | 218.49 | 100.28 | 4.7 | 8.9 | 8.1 | 5.6 | 1.8 | 1.0 | 3.5 |
| **CO2** | 469.90 | 21.72 | 6.3 | 14.2 | 21.3 | 168.05 | 191.00 | 110.85 | 5.7 | 10.7 | 8.8 | 7.1 | 2.2 | 1.2 | 3.2 |
| **CIM PITAMBER** | 246.53 | 22.54 | 2.8 | 9.6 | 11.1 | 76.09 | 118.37 | 52.07 | 5.5 | 9.2 | 8.0 | 6.8 | 2.1 | 1.2 | 4.0 |
| **S.E.** | 18.48 | 0.85 | 0.64 | 0.27 | 1.45 | 14.72 | 20.71 | 10.03 | 0.29 | 0.55 | 0.64 | 0.57 | 0.21 | 0.14 | 0.30 |
| **S.E.D.** | 26.13 | 1.21 | 0.91 | 0.39 | 2.06 | 20.82 | 29.29 | 14.18 | 0.41 | 0.78 | 0.91 | 0.81 | 0.29 | 0.20 | 0.42 |
| **C.D. (P=0.05)** | 64.03 | 2.96 | 2.25 | 0.95 | 5.05 | 51.02 | 71.77 | 34.76 | 1.02 | 1.91 | 2.23 | 1.99 | 0.73 | 0.51 | 1.05 |
| **Note; (DR)** Dry Recovery, **(MRN)** Number of Mother Rhizomes per Plant, **(PRN)** Number of Primary Rhizomes per Plant, **(SRN)** Number of Secondary Rhizomes per Plant, **(MRW)** Weight of Mother Rhizomes per Plant, **(PRW)** Weight of Primary Rhizomes per Plant, **(SRW)** Weight of Secondary Rhizomes per Plant, **(MRL)** Length of Mother Rhizome, **(MRG)** Girth of Mother Rhizome, **(PRL)** Length of Primary Rhizome, **(PRG)** Girth of Primary Rhizome, **(PRD)** Primary Rhizome Diameter, **(PRCD)** Primary Rhizome Core Diameter, and **(SRL)** Length of Secondary Rhizome. | | | | | | | | | | | | | | | |

| **Supplementary Table 3 |** Performance of turmeric germplasm based on curcuminoids content | | | | |
| --- | --- | --- | --- | --- |
| **Germplasm Code No.** | **A**  **(%)** | **B**  **(%)** | **C**  **(%)** | **TCC**  **(%)** |
| **CL-1** | 1.63 | 1.18 | 0.79 | 3.60 |
| **CL-3** | 1.33 | 0.86 | 0.76 | 2.95 |
| **CL-4** | 0.80 | 0.60 | 0.64 | 2.04 |
| **CL-5** | 1.44 | 0.88 | 0.70 | 3.02 |
| **CL-6** | 1.20 | 0.83 | 0.60 | 2.63 |
| **CL-7** | 1.11 | 0.75 | 0.49 | 2.35 |
| **CL-8** | 1.10 | 0.71 | 0.62 | 2.43 |
| **CL-9** | 1.59 | 0.95 | 0.82 | 3.36 |
| **CL-10** | 1.52 | 1.25 | 0.72 | 3.49 |
| **CL-12** | 1.57 | 1.05 | 0.64 | 3.26 |
| **CL-13** | 1.45 | 0.95 | 0.93 | 3.33 |
| **CL-14** | 1.67 | 1.03 | 0.95 | 3.65 |
| **CL-16** | 1.57 | 0.98 | 0.83 | 3.38 |
| **CL-17** | 1.24 | 1.14 | 1.03 | 3.41 |
| **CL-18** | 1.28 | 0.96 | 0.71 | 2.95 |
| **CL-19** | 1.93 | 1.18 | 0.85 | 3.96 |
| **CL-20** | 1.43 | 1.11 | 0.66 | 3.20 |
| **CL-21** | 1.33 | 1.11 | 0.91 | 3.35 |
| **CL-23** | 1.52 | 0.92 | 0.70 | 3.14 |
| **CL-24** | 1.13 | 0.69 | 0.63 | 2.45 |
| **CL-25** | 1.52 | 0.96 | 0.86 | 3.34 |
| **CL-26** | 1.41 | 1.02 | 0.85 | 3.28 |
| **CL-27** | 1.40 | 0.83 | 0.78 | 3.01 |
| **CL-28** | 0.99 | 0.84 | 0.76 | 2.59 |
| **CL-29** | 0.96 | 0.64 | 0.62 | 2.22 |
| **CL-30** | 1.90 | 1.00 | 0.94 | 3.84 |
| **CL-32** | 1.48 | 0.97 | 0.84 | 3.29 |
| **CL-33** | 1.34 | 0.77 | 0.72 | 2.83 |
| **CL-34** | 1.15 | 0.80 | 0.79 | 2.74 |
| **CL-36** | 1.08 | 0.88 | 0.62 | 2.58 |
| **CL-37** | 1.70 | 1.10 | 0.94 | 3.74 |
| **CL-38** | 1.08 | 0.79 | 0.79 | 2.66 |
| **CL-39** | 1.28 | 0.70 | 0.62 | 2.60 |
| **CL-40** | 0.41 | 0.61 | 0.56 | 1.58 |
| **CL-44** | 1.03 | 0.51 | 0.51 | 2.05 |
| **CL-45** | 0.68 | 0.46 | 0.45 | 1.59 |
| **CL-46** | 0.90 | 0.69 | 0.49 | 2.08 |
| **CL-47** | 1.39 | 0.83 | 0.69 | 2.91 |
| **CL-48** | 1.25 | 1.11 | 1.06 | 3.42 |
| **CL-50** | 1.65 | 1.02 | 1.03 | 3.70 |
| **CL-51** | 1.53 | 0.88 | 0.76 | 3.17 |
| **CL-53** | 1.34 | 0.94 | 0.70 | 2.98 |
| **CL-54** | 0.59 | 0.47 | 0.45 | 1.51 |
| **CL-55** | 1.57 | 1.10 | 0.73 | 3.40 |
| **CL-56** | 1.27 | 0.81 | 0.71 | 2.79 |
| **CL-57** | 1.62 | 1.07 | 1.11 | 3.80 |
| **CL-58** | 1.53 | 1.01 | 0.77 | 3.31 |
| **CL-59** | 1.03 | 0.89 | 0.86 | 2.78 |
| **CL-60** | 1.21 | 0.73 | 0.70 | 2.64 |
| **CL-61** | 1.61 | 0.90 | 0.85 | 3.36 |
| **CL-62** | 1.89 | 0.96 | 0.84 | 3.69 |
| **CL-63** | 1.31 | 0.98 | 0.87 | 3.16 |
| **CL-66** | 0.93 | 0.54 | 0.52 | 1.99 |
| **CL-67** | 1.16 | 0.99 | 1.01 | 3.16 |
| **CL-68** | 1.33 | 1.04 | 0.82 | 3.19 |
| **CL-69** | 1.53 | 0.97 | 0.81 | 3.31 |
| **CL-70** | 1.58 | 0.85 | 0.71 | 3.14 |
| **CL-71** | 1.65 | 1.07 | 0.86 | 3.58 |
| **CL-73** | 1.43 | 0.83 | 0.75 | 3.01 |
| **CL-76** | 1.50 | 1.01 | 0.62 | 3.13 |
| **CL-77** | 1.39 | 1.03 | 0.79 | 3.21 |
| **CL-79** | 1.19 | 0.78 | 0.78 | 2.75 |
| **CL-80** | 0.76 | 0.73 | 0.65 | 2.14 |
| **CL-82** | 1.63 | 0.87 | 0.72 | 3.22 |
| **CL-83** | 0.71 | 0.82 | 0.69 | 2.22 |
| **CL-84** | 1.49 | 0.94 | 0.77 | 3.20 |
| **CL-86** | 1.59 | 1.10 | 0.75 | 3.44 |
| **CL-87** | 1.55 | 0.98 | 0.85 | 3.38 |
| **CL-90** | 0.89 | 0.80 | 0.60 | 2.29 |
| **CL-91** | 1.53 | 0.84 | 0.86 | 3.23 |
| **CL-92** | 1.42 | 1.06 | 0.70 | 3.18 |
| **CL-93** | 1.57 | 0.93 | 0.65 | 3.15 |
| **CL-94** | 1.28 | 0.74 | 0.74 | 2.76 |
| **CL-95** | 1.18 | 0.95 | 0.85 | 2.98 |
| **CL-96** | 1.62 | 0.93 | 0.83 | 3.38 |
| **CL-97** | 1.64 | 0.92 | 0.71 | 3.27 |
| **CL-98** | 1.21 | 0.82 | 0.77 | 2.80 |
| **CL-99** | 1.76 | 0.99 | 0.78 | 3.53 |
| **CL-101** | 1.55 | 0.92 | 0.74 | 3.21 |
| **CL-102** | 1.39 | 0.88 | 0.77 | 3.04 |
| **CL-103** | 1.88 | 1.13 | 0.92 | 3.93 |
| **CL-104** | 1.46 | 0.83 | 0.66 | 2.95 |
| **CL-105** | 0.87 | 0.67 | 0.75 | 2.29 |
| **CL-106** | 0.53 | 0.38 | 0.40 | 1.31 |
| **CL-107** | 1.33 | 1.00 | 0.74 | 3.07 |
| **CL-109** | 1.37 | 0.84 | 0.77 | 2.98 |
| **CL-110** | 1.52 | 0.97 | 0.72 | 3.21 |
| **CL-111** | 0.65 | 0.68 | 0.56 | 1.89 |
| **CL-112** | 1.31 | 0.78 | 0.69 | 2.78 |
| **CL-113** | 1.09 | 0.84 | 0.77 | 2.70 |
| **CL-115** | 1.32 | 0.92 | 0.61 | 2.85 |
| **CL-116** | 1.50 | 0.93 | 0.80 | 3.23 |
| **CL-117** | 0.52 | 0.58 | 0.67 | 1.77 |
| **CL-118** | 1.38 | 0.83 | 0.69 | 2.90 |
| **CL-119** | 1.90 | 1.08 | 0.87 | 3.85 |
| **CL-123** | 1.77 | 1.03 | 0.84 | 3.64 |
| **CL-124** | 1.10 | 0.62 | 0.61 | 2.33 |
| **CL-125** | 1.49 | 0.83 | 0.76 | 3.08 |
| **CL-126** | 1.64 | 0.90 | 0.72 | 3.26 |
| **CL-127** | 0.58 | 0.63 | 0.58 | 1.79 |
| **CL-128** | 1.63 | 1.06 | 1.01 | 3.70 |
| **CL-129** | 0.89 | 0.63 | 0.55 | 2.07 |
| **CL-136** | 0.70 | 0.57 | 0.64 | 1.91 |
| **CL-137** | 1.06 | 0.73 | 0.75 | 2.54 |
| **CL-138** | 1.14 | 0.74 | 0.65 | 2.53 |
| **CL-139** | 1.47 | 0.84 | 0.66 | 2.97 |
| **CL-140** | 1.34 | 0.74 | 0.61 | 2.69 |
| **CL-141** | 1.15 | 0.71 | 0.56 | 2.42 |
| **CL-143** | 1.03 | 0.79 | 0.63 | 2.45 |
| **CL-145** | 0.88 | 0.57 | 0.46 | 1.91 |
| **CL-153** | 1.39 | 0.76 | 0.71 | 2.86 |
| **CL-154** | 1.26 | 0.68 | 0.64 | 2.58 |
| **CL-155** | 1.03 | 0.51 | 0.51 | 2.05 |
| **CL-157** | 2.17 | 1.31 | 1.07 | 4.55 |
| **CL-159** | 1.07 | 0.75 | 0.68 | 2.50 |
| **CL-161** | 1.62 | 0.90 | 0.77 | 3.29 |
| **CL-162** | 0.93 | 0.65 | 0.76 | 2.34 |
| **CL-163** | 1.50 | 0.97 | 0.74 | 3.21 |
| **CL-164** | 1.63 | 0.81 | 0.83 | 3.27 |
| **CL-165** | 1.44 | 0.87 | 0.66 | 2.97 |
| **CL-166** | 1.90 | 1.07 | 0.90 | 3.87 |
| **CL-167** | 1.41 | 0.78 | 0.62 | 2.81 |
| **CL-168** | 1.75 | 1.07 | 0.79 | 3.61 |
| **CL-171** | 1.63 | 0.88 | 0.75 | 3.26 |
| **CL-176** | 1.61 | 0.81 | 0.66 | 3.08 |
| **CL-177** | 1.42 | 0.72 | 0.74 | 2.88 |
| **CL-178** | 1.43 | 1.00 | 0.90 | 3.33 |
| **CL-179** | 1.48 | 0.81 | 0.61 | 2.90 |
| **CL-180** | 1.93 | 1.09 | 0.89 | 3.91 |
| **CL-181** | 1.74 | 1.05 | 0.85 | 3.64 |
| **CL-182** | 1.63 | 0.74 | 0.69 | 3.06 |
| **CL-183** | 1.58 | 0.82 | 0.74 | 3.14 |
| **CL-186** | 1.95 | 1.11 | 1.04 | 4.10 |
| **CL-188** | 1.48 | 1.13 | 0.84 | 3.45 |
| **CL-191** | 1.66 | 0.84 | 0.79 | 3.29 |
| **CL-193** | 1.83 | 1.04 | 0.83 | 3.70 |
| **CL-196** | 1.36 | 0.83 | 0.61 | 2.80 |
| **CL-197** | 1.79 | 1.06 | 0.81 | 3.66 |
| **CL-202** | 1.18 | 0.70 | 0.57 | 2.45 |
| **CL-203** | 1.30 | 0.82 | 0.65 | 2.77 |
| **CL-204** | 1.60 | 0.95 | 0.72 | 3.27 |
| **CL-205** | 1.52 | 0.83 | 0.75 | 3.10 |
| **CL-206** | 1.29 | 0.97 | 0.95 | 3.21 |
| **CL-207** | 1.40 | 0.90 | 0.81 | 3.11 |
| **CL-208** | 1.52 | 0.89 | 0.76 | 3.17 |
| **CL-210** | 1.63 | 0.92 | 0.70 | 3.25 |
| **CL-211** | 1.86 | 1.17 | 0.74 | 3.77 |
| **CL-214** | 1.75 | 0.87 | 0.77 | 3.39 |
| **CL-215** | 1.20 | 0.69 | 0.64 | 2.53 |
| **CL-216** | 1.42 | 1.18 | 1.24 | 3.84 |
| **CL-217** | 1.31 | 0.92 | 0.70 | 2.93 |
| **CL-218** | 1.28 | 0.78 | 0.59 | 2.65 |
| **CL-219** | 1.29 | 0.96 | 0.67 | 2.92 |
| **CL-220** | 1.48 | 1.04 | 0.85 | 3.37 |
| **CL-221** | 1.52 | 0.88 | 0.86 | 3.26 |
| **CL-222** | 1.11 | 0.78 | 0.55 | 2.44 |
| **CL-223** | 1.35 | 0.87 | 0.78 | 3.00 |
| **CL-224** | 1.03 | 0.66 | 0.66 | 2.35 |
| **CL-225** | 1.72 | 1.15 | 0.70 | 3.57 |
| **CL-226** | 1.29 | 0.91 | 0.76 | 2.96 |
| **CL-227** | 1.59 | 1.00 | 0.94 | 3.53 |
| **CL-229** | 0.41 | 0.48 | 0.37 | 1.26 |
| **CL-231** | 1.31 | 0.86 | 0.70 | 2.87 |
| **CL-232** | 1.19 | 0.91 | 0.77 | 2.87 |
| **CL-233** | 1.15 | 0.75 | 0.61 | 2.51 |
| **CL-234** | 1.00 | 0.80 | 0.55 | 2.35 |
| **CL-235** | 1.22 | 0.82 | 0.58 | 2.62 |
| **CL-236** | 1.33 | 0.83 | 0.60 | 2.76 |
| **CL-237** | 1.46 | 0.96 | 0.79 | 3.21 |
| **CL-238** | 2.08 | 1.28 | 0.94 | 4.30 |
| **CL-239** | 1.37 | 0.89 | 0.70 | 2.96 |
| **CL-240** | 1.27 | 0.95 | 0.80 | 3.02 |
| **CL-241** | 1.75 | 1.05 | 0.82 | 3.62 |
| **CL-242** | 1.75 | 1.22 | 0.92 | 3.89 |
| **CL-243** | 1.61 | 0.92 | 0.73 | 3.26 |
| **CL-244** | 1.17 | 0.93 | 0.84 | 2.94 |
| **CL-245** | 1.39 | 0.81 | 0.88 | 3.08 |
| **CL-246** | 1.60 | 0.96 | 0.79 | 3.35 |
| **CL-247** | 1.72 | 1.05 | 0.73 | 3.50 |
| **CL-248** | 1.25 | 0.93 | 0.74 | 2.92 |
| **CL-249** | 1.51 | 0.99 | 0.67 | 3.17 |
| **CL-250** | 1.77 | 1.10 | 0.91 | 3.78 |
| **CL-251** | 1.70 | 1.03 | 0.85 | 3.58 |
| **CL-252** | 0.89 | 0.66 | 0.54 | 2.09 |
| **CL-253** | 1.81 | 1.45 | 0.96 | 4.22 |
| **CL-254** | 1.41 | 0.92 | 0.76 | 3.09 |
| **CL-256** | 1.29 | 0.73 | 0.64 | 2.66 |
| **CL-257** | 1.96 | 0.91 | 0.85 | 3.72 |
| **CL-258** | 2.10 | 1.24 | 0.89 | 4.23 |
| **CL-261** | 1.53 | 0.91 | 0.69 | 3.13 |
| **CL-265** | 0.74 | 0.52 | 0.39 | 1.65 |
| **CL-266** | 0.83 | 0.67 | 0.66 | 2.16 |
| **CL-267** | 1.63 | 1.05 | 0.75 | 3.43 |
| **CL-268** | 1.17 | 0.95 | 0.61 | 2.73 |
| **CL-270** | 1.43 | 0.92 | 0.94 | 3.29 |
| **CL-271** | 1.27 | 0.82 | 0.71 | 2.80 |
| **CL-272** | 2.13 | 1.13 | 1.11 | 4.37 |
| **CL-273** | 1.60 | 0.94 | 0.73 | 3.27 |
| **CL-274** | 0.99 | 0.66 | 0.55 | 2.20 |
| **CL-275** | 1.13 | 1.08 | 0.83 | 3.04 |
| **CO2** | 1.58 | 0.97 | 0.82 | 3.37 |
| **CIM PITAMBER** | 1.95 | 1.10 | 0.88 | 3.92 |
| **S.E** | 0.02 | 0.02 | 0.03 | 0.03 |
| **S.E.D** | 0.03 | 0.03 | 0.05 | 0.04 |
| **C.D (P=0.05)** | 0.07 | 0.08 | 0.12 | 0.09 |
| **Note; (A)** Curcumin, **(B)** Bisdemethoxycurcumin, **(C)** Bisdemethoxycurcumin, and **(TCC)** Total Curcuminoid Content. | | | | |
